# Sodium Selenite Preconditioning Reprograms Mesenchymal Stem Cell Behavior Under Inflammatory Stress to Enhance Anti-Inflammatory Macrophage Responses

**DOI:** 10.1007/s12011-026-05192-5

**Published:** 2026-07-03

**Authors:** Iolanda Silva Rafael Pizzolato-Cezar, Edson Naoto Makiyama, Luis Ricardo Pizzolato-Cezar, Kalil Auzier Martins Costa, Carlos Eduardo da Silva Gonçalves, Sumara de Freitas, Renata Chaves Albuquerque, Ricardo Ambrósio Fock

**Affiliations:** 1 Laboratory of Experimental Hematology, Department of Clinical and Toxicological Analysis, School of Pharmaceutical Sciences, University of São Paulo, Lineu Prestes Avenue, 580 – Block17, São Paulo, SP 05508-900 Brazil; 2 Department of Biochemistry, Institute of Chemistry, University of São Paulo, São Paulo, Brazil

**Keywords:** Mesenchymal Stem Cells, Selenium, Immunomodulation, Macrophages, Inflammation

## Abstract

Inflammation critically influences mesenchymal stem cell (MSC) behavior, affecting their survival, plasticity, and immunomodulatory functions. MSC preconditioning has emerged as a promising strategy to enhance their therapeutic potential before clinical application. Among the factors capable of modulating MSC responses, selenium has gained attention due to its role in regulating inflammatory signaling. In this study, we investigated whether high-dose sodium selenite (Na_2_SeO_3_) preconditioning modulates MSC behavior under inflammatory stimulation and how these changes affect macrophage responses through paracrine mechanisms. MSCs were preconditioned with Na_2_SeO_3_ in the presence or absence of lipopolysaccharide (LPS). Cellular metabolism, apoptosis, cell-cycle progression, migration, intracellular signaling, reactive oxygen species (ROS) production, and soluble mediator release were evaluated. Macrophage responses were assessed using conditioned medium from treated MSCs. Na_2_SeO_3_ alone did not significantly affect MSC metabolic viability. However, under inflammatory conditions, Na_2_SeO_3_ enhanced apoptotic signaling and promoted cell-cycle accumulation in S/G2/M phases. In addition, Na_2_SeO_3_ preconditioning reduced intracellular ROS levels, attenuated NFκB and JNK activation, and altered the MSC secretory profile, increasing nitric oxide, transforming growth factor-β, and prostaglandin E2 production while decreasing IL-6 levels. Importantly, conditioned medium from preconditioned MSCs reprogrammed macrophage responses, reducing TNF-α and IL-1β production while increasing IL-1 receptor antagonist levels. These findings demonstrate that high-dose Na_2_SeO_3_ reprograms MSCs under inflammatory stress, enhancing their anti-inflammatory paracrine activity and capacity to modulate macrophage inflammatory responses. These results identify selenium as a key regulator of MSC-mediated immunomodulation and support sodium selenite preconditioning as a strategy to optimize MSC-based therapies for inflammatory diseases.

**Supplementary Information:**

The online version contains supplementary material available at 10.1007/s12011-026-05192-5.

## Introduction

Inflammation is a fundamental biological response triggered by tissue injury, infection, or metabolic imbalance, and its proper regulation is essential for maintaining tissue homeostasis [1, 2]. Dysregulated or chronic inflammation contributes to the progression of several pathological conditions, including autoimmune diseases, cancer, cardiovascular disorders, and neurodegeneration [1, 2]. In this context, mesenchymal stem cells (MSCs) have emerged as key regulators of inflammatory processes due to their potent immunomodulatory and anti-inflammatory properties [3–5]. Physiologically, these cells are primarily involved in tissue repair processes, cell replacement, and modulation of the immune system [5, 6].

MSCs actively participate in the control of immune responses by interacting with both innate and adaptive immune cells [5, 7, 8]. These cells are capable of reducing excessive inflammatory reactions through the secretion of soluble mediators, cytokines, chemokines, and growth factors that modulate immune cell activation and function [4, 5, 9]. Experimental studies have demonstrated that MSC administration attenuates inflammatory responses and promotes tissue repair in a wide range of experimental disease models [3–5]. Following systemic or local administration, MSCs are capable of sensing inflammatory chemokine gradients and migrating toward sites of tissue injury and inflammation [5, 9, 10]. At these sites, MSCs exert their therapeutic effects predominantly through paracrine and immunomodulatory mechanisms rather than direct tissue replacement [5, 6, 10]. Through the secretion of cytokines, growth factors, extracellular vesicles, and other bioactive mediators, MSCs regulate immune cell activation, suppress excessive inflammatory responses, and support tissue regeneration and restoration of microenvironmental homeostasis [5, 9, 10]. However, exposure of MSCs to persistent inflammatory conditions may impair their survival, secretory profile, and immunomodulatory capacity, potentially limiting their therapeutic efficacy [10–12]. Therefore, strategies capable of preserving or enhancing MSC functionality under inflammatory stress have become increasingly relevant.

Importantly, the immunomodulatory activity of MSCs is highly dependent on signals derived from the surrounding inflammatory microenvironment, which dynamically shapes their functional phenotype and secretory profile [5, 7, 10]. This biological plasticity has stimulated increasing interest in strategies aimed at functionally optimizing MSCs before therapeutic administration. In this context, MSC preconditioning has emerged as a promising approach in regenerative medicine and cell therapy [11, 12]. MSC preconditioning refers to the controlled in vitro exposure of these cells to specific stimuli, including inflammatory mediators, hypoxia, metabolic stressors, antioxidants, or pharmacological compounds, with the aim of enhancing their immunomodulatory, anti-inflammatory, regenerative, and paracrine properties prior to transplantation [12, 13]. This strategy has been extensively investigated as a means to improve MSC therapeutic efficacy by modulating inflammatory signaling pathways, survival capacity, migratory potential, and secretory activity [11–13]. Importantly, the purpose of MSC preconditioning is not the systemic administration of the preconditioning agent itself, but rather the ex vivo functional modulation of MSCs before their potential therapeutic application [11–13].

In this context, trace elements have emerged as important modulators of immune and cellular homeostasis. These micronutrients play essential roles in maintaining immune homeostasis and regulating cellular responses to stress and infection by acting as cofactors for key enzymes, influencing signaling pathways, and modulating the production of cytokines and other immune mediators [14, 15]. Among these elements, selenium has gained increasing attention due to its essential role in immune and inflammatory regulation, since both deficiency and excess can disrupt immune function, highlighting its critical importance in balancing protective immune responses while preventing excessive inflammation [16–18].

Altogether, current evidence indicates that selenium may influence MSC functions [18, 19]; however, the molecular mechanisms underlying the interaction between selenium metabolism and MSC-mediated inflammatory regulation remain incompletely understood. Considering the growing interest in MSC preconditioning strategies, the present study investigated the molecular responses of MSCs to lipopolysaccharide (LPS)-induced inflammatory stimulation and evaluated whether sodium selenite-mediated preconditioning could modulate the inflammatory and immunomodulatory properties of MSCs, particularly in the context of MSC–macrophage interactions under inflammatory conditions.

## Materials and methods

### Cell Culture of MSCs

Cell culture was performed using the murine mesenchymal stem cells (MSCs) C3H10T1/2 cell line, which exhibits typical mesenchymal stromal characteristics, including plastic adherence and fibroblast-like morphology [20, 21]. C3H10T1/2 cells were originally derived from embryonic tissue of C3H mice and were obtained from the American Type Culture Collection (Clone 8, ATCC^®^ CCL-226™, USA). Owing to their multipotent differentiation potential and stromal phenotype, this cell line is widely used as an in vitro model of mesenchymal stem/stromal cells [20, 21]. Cells were cultured in Dulbecco’s Modified Eagle Medium (DMEM) supplemented with 10% fetal bovine serum (FBS), 2 g/L HEPES buffer, 100 IU/mL sodium penicillin G (Sigma-Aldrich, USA), and 100 µg/mL streptomycin (Sigma-Aldrich, USA), with the final pH adjusted to 7.2–7.4. Cell cultures were maintained at 37 °C in a humidified atmosphere containing 5% CO_2_. Cells were initially seeded in 25 cm^2^ culture flasks and subsequently expanded to 75 cm^2^ flasks. For experiments, cells were plated at a density of 1 × 10^6^ cells/mL and grown until approximately 70% confluence to ensure optimal proliferation conditions.

### Measurement of Metabolic activity in MSCs treated with Na_2_SeO_3_

Cellular metabolic activity was assessed using the colorimetric MTT assay, which measures the reduction of MTT (3-(4,5-dimethylthiazol-2-yl)-2,5-diphenyltetrazolium bromide) into blue–purple formazan crystals by metabolically active cells [22]. MSCs were seeded in 12-well plates at a density of 1 × 10^6^ cells/mL and cultured in DMEM (as previously described) for 24 h. Cells were then treated with Na_2_SeO_3_ at different concentrations (0, 0.625, 1.25, 2.5, 5, 10, 20, 40, 80 and 160 µM). After 24 h of treatment, the culture medium was replaced with 900 µL of DMEM supplemented with 10% FBS and 100 µL of MTT solution (5 mg/mL in PBS) (Sigma-Aldrich, USA). Cells were incubated for 4 h at 37 °C in a humidified atmosphere with 5% CO_2_. Subsequently, the medium was removed and 1000 µL of dimethyl sulfoxide (DMSO) was added to dissolve the formazan crystals. Plates were incubated under gentle agitation for 30 min at 37 °C. Absorbance was measured at 570 nm using a Varioskan Flash spectrophotometer (Thermo Scientific, UK).

### Experimental Design and Treatment Groups

MSCs were divided into four experimental groups: (*i*) control, (*ii*) Na_2_SeO_3_-treated, (*iii*) LPS-stimulated, and (*iv*) LPS + Na_2_SeO_3_-treated. To mimic an inflammatory microenvironment, MSCs were stimulated with LPS, an endotoxin derived from the cell wall of Gram-negative bacteria, at a concentration of 1.25 µg/mL [23, 24], and treated with Na_2_SeO_3_ at a concentration of 20 µM. The Na_2_SeO_3_ concentration was selected based on a concentration–response curve generated by the MTT assay after 24 h of exposure, followed by IC50 determination (Figure S**1**). The 20 µM concentration corresponded to the highest dose that did not significantly reduce MSC metabolic activity and was therefore selected for subsequent experiments.

### Flow Cytometric Analysis of proliferation, Cell Viability and Cell Cycle in MSCs

MSCs were seeded in 6-well plates at a density of 1 × 10^6^ cells/mL and incubated for 24 h under the respective experimental conditions, as described previously. After treatment, adherent cells were detached using 200 µL of trypsin, and enzymatic activity was neutralized by adding 300 µL of DMEM supplemented with 10% fetal bovine serum (FBS).

For viability and cell death analysis, cell suspensions were transferred to acrylic tubes and centrifuged at 1800 rpm for 10 min at 25 °C. The supernatant was discarded, and the cell pellet was washed with 1000 µL of phosphate-buffered saline (PBS) to remove residual trypsin, followed by centrifugation under the same conditions. After washing, samples were labeled according to their respective experimental groups. The percentage of viable cells and the occurrence of early apoptosis, late apoptosis, and necrosis were evaluated by flow cytometry using Annexin V and propidium iodide (PI) staining. Reagents were added according to the manufacturer’s instructions (BD Pharmingen, USA), including 50 µL of annexin binding buffer, 2.5 µL of Annexin V, and 8 µL of PI. For the negative control (non-viable cells), 50 µL of ethanol was added. Samples were incubated for 30 min at room temperature, protected from light. Data acquisition was performed using a FACSCanto II flow cytometer (Becton Dickinson, USA), with a total of 20,000 events collected per sample. Data analysis was conducted using FlowJo software (Becton Dickinson, USA).

For cell cycle analysis, cells were processed as described above and resuspended after PBS washing and centrifugation. Reagents were then added to each tube, including 200 µL of Triton X-100 (0.1%), sodium citrate (0.1%) diluted in PBS, 20 µL of RNase (4 mg/mL), and 8 µL of propidium iodide (PI). Samples were incubated in a water bath at 37 °C for 30 min, protected from light, to ensure optimal RNase activity. Subsequently, samples were analyzed by flow cytometry using a FACSCanto II flow cytometer (Becton Dickinson, USA). The percentages of cells in the G_0_/G_1_ and S/G_2_/M phases were determined using FlowJo software (Becton Dickinson, USA).

### Assessment of Soluble Factor Secretion by MSCs

MSCs were cultured in 6-well plates at a density of 1 × 10^6^ cells/mL for 24 h at 37 °C in a humidified atmosphere containing 5% CO_2_, under the respective treatments described previously. After incubation, the supernatants were collected and stored at − 40 °C for subsequent quantification of soluble factors by enzyme-linked immunosorbent assay (ELISA) and for immunomodulatory assays as described below. The concentrations of the following cytokines and growth factors were determined using ELISA kits (R&D Systems, Minneapolis, MN, USA): interleukin-6 (IL-6; DY406), interleukin-1 beta (IL-1β; DY401), transforming growth factor beta (TGF-β; DY1679), hepatocyte growth factor (HGF; DY2207), tumor necrosis factor alpha (TNF-α; DY410), and interleukin-1 receptor antagonist (IL-1ra; DY480). Additionally, prostaglandin E2 (PGE_2_; 514010, Cayman Chemical Co., Ann Arbor, MI, USA) levels were quantified according to the manufacturer’s instructions. Nitric oxide (NO) levels were quantified using the Griess reaction [25], a colorimetric method that converts NO^•^ present in the samples into nitrite (NO_2_⁻). Adherent cells were washed with PBS and used for protein expression analysis by western blotting.

### Measurement of intracellular ROS production

Intracellular reactive oxygen species (ROS) production was evaluated by flow cytometry using the fluorescent probe 2’,7’-dichlorodihydrofluorescein diacetate (DCFH-DA). For the experiments, 1 × 10^6^ cells were seeded into 6-well plates containing DMEM supplemented with 10% fetal bovine serum (FBS). After cell adhesion, cells were pretreated with N-acetyl-L-cysteine (NAC; 5 mM; Sigma-Aldrich, A7250) [26] for 1 h, followed by treatment with sodium selenite (20 µM) and stimulation with LPS, and then incubated for 24 h at 37 °C in a humidified atmosphere containing 5% CO_2_.

After the incubation period, cells were incubated with DCFH-DA (10 µM; Sigma-Aldrich, D6883) in DMEM containing 1% FBS for 30 min at 37 °C in the dark. Subsequently, cells were washed with PBS and resuspended in PBS for flow cytometry acquisition. Data acquisition was performed using a BD FACSCanto II flow cytometer (Becton Dickinson, USA), with 20,000 events acquired per sample. Data analysis was performed using FlowJo software (Becton Dickinson, USA), and results were expressed as median FITC fluorescence intensity.

### Protein Expression Analysis by Western Blotting

The expression levels of NFκB p65, JNK, Cdc42, RhoA and Rac1/2/3 in MSCs were analyzed by western blotting. As described above, after supernatant collection, adherent cells were washed with PBS, and the 6-well plates were placed on ice for 5 min. Cells were then subjected to sonication for 30 s to obtain total cell lysates. Protein extraction was performed using RIPA buffer (89901; Thermo Scientific, USA) supplemented with 100 µL protease inhibitor cocktail (S8820; Sigma-Aldrich, USA), 10 µL PMSF (1%, 0.1 M), and 50 µL NaF (5%, 1 M), resulting in a final volume of 1000 µL.

The supernatant was collected into labeled microtubes, homogenized by vortexing for 1 min, and centrifuged at 14,000 rpm for 10 min at 4 °C. After centrifugation, 100 µL of the supernatant was mixed with 25 µL of 5X Laemmli buffer containing 100 mM Tris-HCl (pH 6.8), 10% SDS, 50% glycerol, and 0.1% bromophenol blue. Samples were heated at 100 °C for 10 min to denature the proteins. Protein concentration was determined using the Pierce™ BCA Protein Assay Kit (Thermo Scientific, USA), according to the manufacturer’s instructions. A total of 20 µg of protein per sample was loaded onto 10% SDS-polyacrylamide gels and transferred onto nitrocellulose membranes (Millipore, USA). Membranes were blocked with 5% BSA in TBST (Tris-buffered saline containing Tween 20) for 2 h under agitation and then incubated overnight at 4 °C with the following primary antibodies: phospho-nuclear factor kappa B (p-NFκB; 1:1000; Cell Signaling #3031), nuclear factor kappa B (NFκB; 1:1000; Cell Signaling #4764), phospho-c-Jun N-terminal kinase (p-JNK; 1:1000; Cell Signaling #4668), c-Jun N-terminal kinase (JNK; 1:1000; Cell Signaling #3708), cell division control protein 42 (Cdc42; 1:500; Santa Cruz sc-8401), Ras homolog family member A (RhoA; 1:1000; Santa Cruz sc-418), Ras-related C3 botulinum toxin substrate 1/2/3 (Rac1/2/3; 1:1000; Santa Cruz sc-514583), and β-actin (1:50,000; Sigma-Aldrich A3854). After three washes with TBST, membranes were incubated with the appropriate HRP-conjugated secondary antibodies: anti-rabbit IgG (1:1000; Cell Signaling #7074) or anti-mouse IgG (1:5000; BD Biosciences 554002) for 2 h. Protein bands were visualized using enhanced chemiluminescence (ECL Prime Amersham™ detection kit; Cytiva, UK) and images were captured with the ImageQuantTM 400^®^ system (GE Healthcare, USA). Band intensities were quantified using ImageQuant TL^®^ software (GE Healthcare, USA) and normalized to β-actin as a loading control.

### Cell Migration Assay – Scratch Wound Healing Assay

To evaluate migratory potential, MSCs were seeded in 6-well plates and cultured for 24 h in DMEM supplemented with 10% fetal bovine serum (FBS) until reaching approximately 90–100% confluence. After incubation, the wells were gently washed with PBS to remove detached cells, and the medium was replaced with fresh DMEM containing 10% FBS in order to eliminate previously secreted soluble factors that could influence migration and indirectly affect cell proliferation. Subsequently, the same experimental groups and treatments described previously were applied, and a linear scratch was generated across the cell monolayer using a sterile 200 µL pipette tip (P200). The same field region was monitored over time, and images were acquired at 0 h, 30 min, 1 h, 2 h, 4 h, 6 h, 12 h and 24 h. The wound area was quantified at each time point using ImageJ software (National Institutes of Health, USA).

### Macrophage Culture for Evaluation of MSCs Immunomodulatory Effects

Selected immunomodulatory effects of MSCs were evaluated using conditioned medium collected from MSCs cultures, as previously described, which was applied to LPS-stimulated macrophages for 24 h. For this purpose, the murine mono-macrophage cell line RAW 264.7, obtained from the American Type Culture Collection (ATCC^®^, TIB-71™, USA), was used.

RAW 264.7 cells were initially expanded in Dulbecco’s Modified Eagle Medium (DMEM) and maintained at 37 °C in a humidified atmosphere containing 5% CO_2_. The culture medium was replaced every 3 days, and when cultures reached approximately 70% confluence, cells were detached by scraping using a cell scraper and subsequently used for the experiments.

RAW 264.7 cells were seeded in 24-well plates at a density of 1 × 10^6^ cells/mL, stimulated with LPS (1.25 µg/mL) [23, 24] and incubated for 24 h in the presence or absence of MSCs-conditioned medium to evaluate the effects of MSCs-derived soluble factors. Following incubation, the levels of the following cytokines were quantified: interleukin-1 beta (IL-1β; DY401), tumor necrosis factor alpha (TNF-α; DY410), and interleukin-1 receptor antagonist (IL-1ra; DY480). All measurements were performed using DuoSet ELISA kits (R&D Systems, Minneapolis, MN, USA) according to the manufacturer’s instructions. Additionally, the expression levels of nuclear factor kappa B (NFκB) were analyzed by Western blot in macrophages, following the methodology described above.

### Statistical Analysis

Results were analyzed using two-way analysis of variance (ANOVA) and Tukey’s post hoc test. For concentration determination in the MTT assay, data were analyzed using one-way ANOVA followed by Dunnett’s post hoc test. Results were considered statistically significant at a 5% significance level (*p* < 0.05). Statistical analyses were performed using GraphPad Prism^®^ software (GraphPad Software Inc., USA).

## Results

### Effect of Na_2_SeO_3_ on the Metabolism, Viability, and Cell Cycle of MSCs

**Fig. 1:**
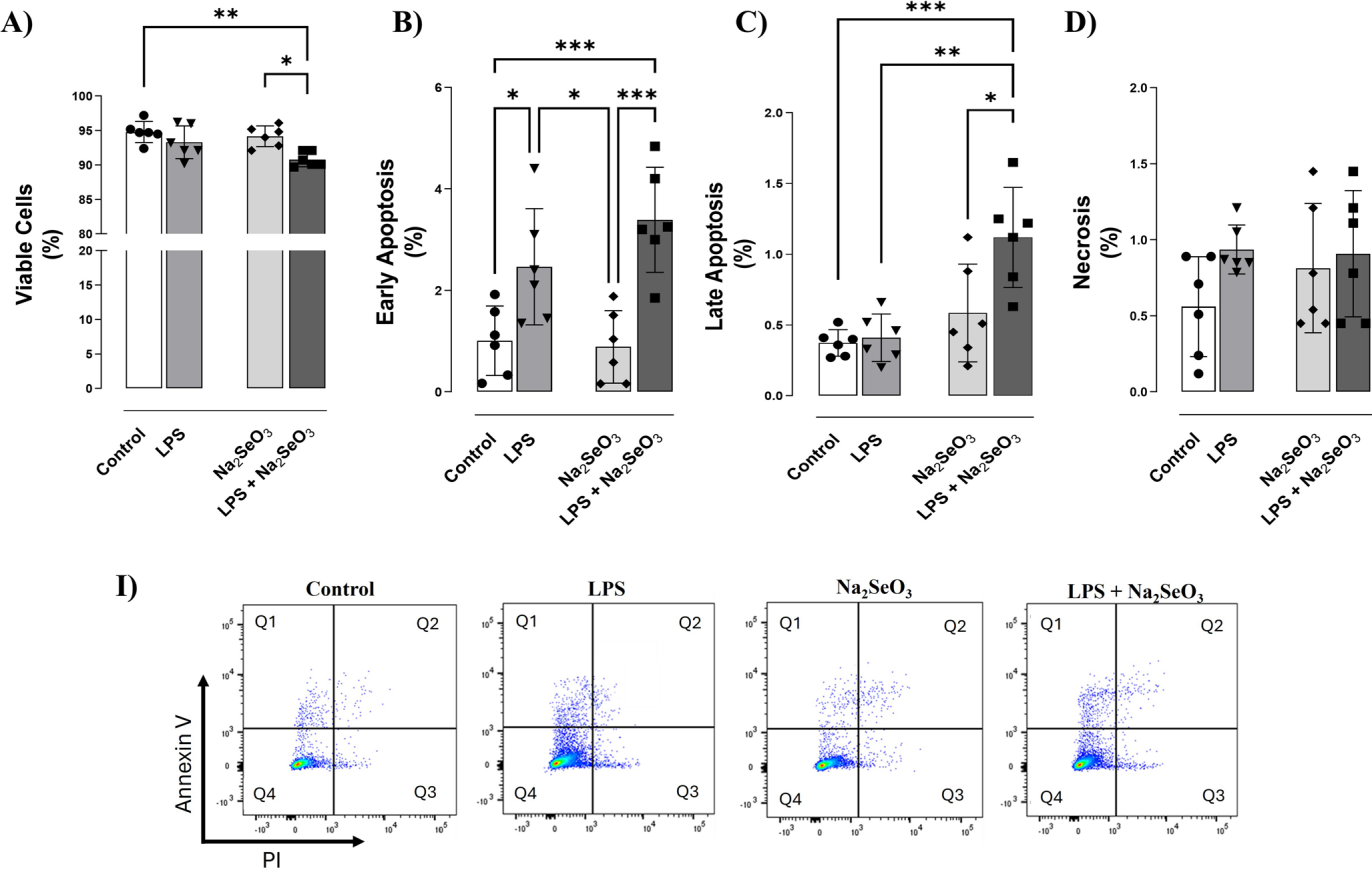
Analysis of the effects of Na_2_SeO_3_ and LPS on MSCs. The percentages of (**A**) viable cells, (**B**) early apoptosis, (**C**) late apoptosis, and (**D**) necrotic cells were then evaluated. Data are expressed as mean ± SD. (**I**) Representative flow cytometry analysis of apoptosis and necrosis using Annexin V and propidium iodide (PI) staining. Q1 indicates early apoptosis, Q2 late apoptosis, Q3 necrosis, and Q4 viable cells. *n* = 6. (*p < 0.05; **p < 0.01 and ***p < 0.001)

To determine the experimental concentration of Na_2_SeO_3_, the mitochondrial metabolic activity of MSCs was assessed using the MTT assay at different compound concentrations (Figure S1). Among the tested doses, a significant reduction in metabolic activity was observed at higher Na_2_SeO_3_ concentrations (above 40 µM), indicating a dose-dependent cytotoxic effect in these cells (Figure S1). Based on these findings, 20 µM was selected for subsequent experiments, as it represented the highest concentration that did not significantly reduce MSC metabolic activity or overall cell viability after 24 h of exposure. This concentration was therefore considered appropriate to evaluate the effects of sodium selenite-mediated preconditioning on MSC functional and immunomodulatory responses under inflammatory stimulation while preserving overall cell viability. Additionally, although the percentage of viable cells remained above 90% in all experimental groups (Fig. 1A), flow cytometry analysis revealed that combined treatment (LPS + Na_2_SeO_3_) significantly increased the percentage of cells undergoing early apoptosis, an effect that was also observed for late apoptosis (Fig. 1B and **C**, respectively). In contrast, no statistically significant differences were detected in the percentage of necrotic cells among the experimental groups (Fig. 1D).

**Fig. 2:**
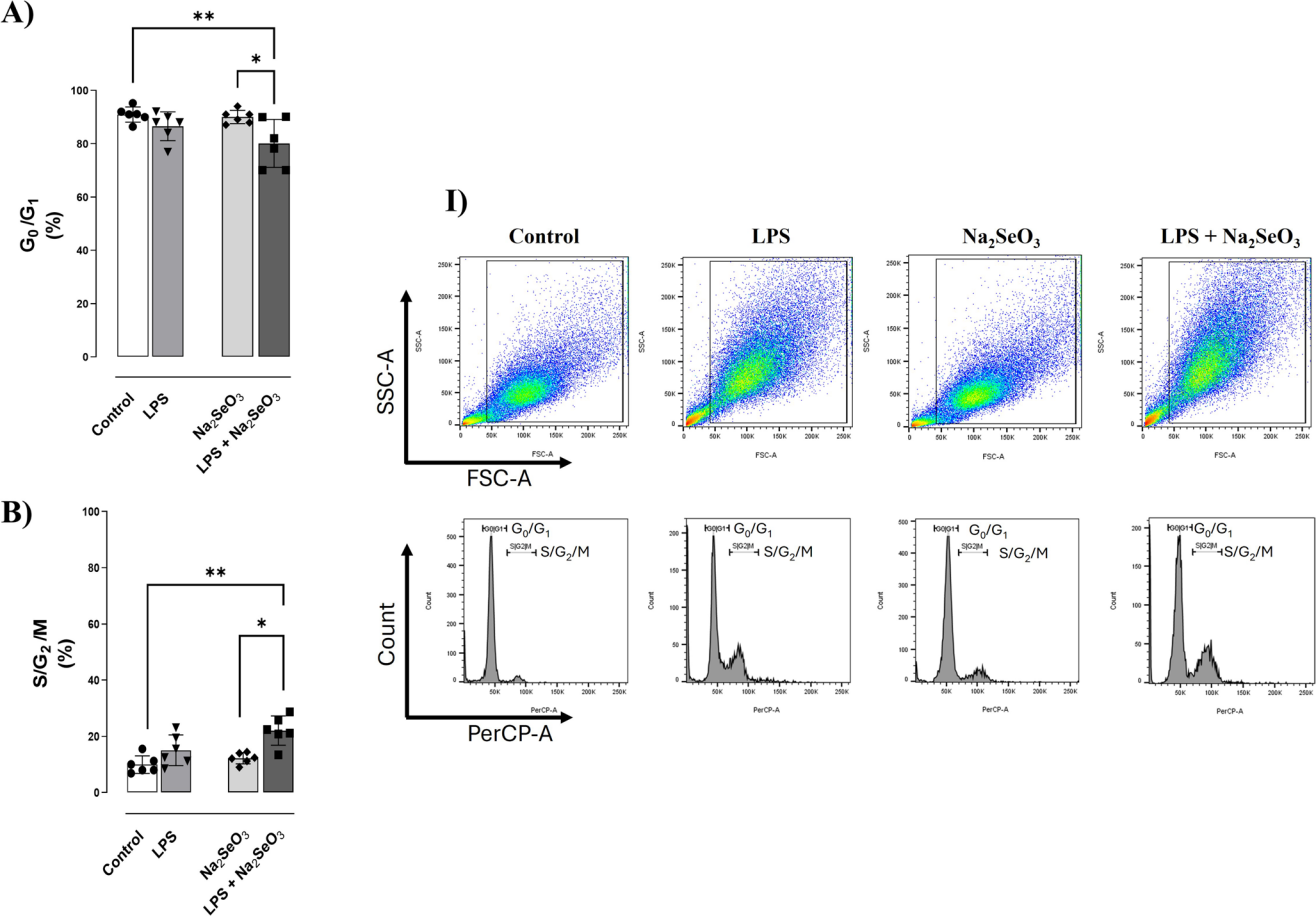
Analysis of the effects of Na_2_SeO_3_ and LPS on MSC cell cycle progression. **(A)** Percentage of cells in the G0/G1 phase. **(B)** Percentage of cells in the S/G2/M phases. **(C)** Cell proliferation. Data are expressed as mean ± SD. (**I**) Representative analysis of MSC cell cycle distribution. *n* = 6. (*p < 0.05 and **p < 0.01)

Cell cycle analysis (Fig. 2A and **B**) demonstrated that approximately 90% of MSCs remained in the G_0_/G_1_ phase, indicating a predominantly quiescent profile. Conversely, a significant increase in the cell population in the S/G_2_/M phases was observed in the LPS + Na_2_SeO_3_–treated group, suggesting that the combined stimuli potentiate cell cycle accumulation in these phases (Fig. 2B).

### Na_2_SeO_3_ Modulates NFκB and JNK Activation in MSCs under LPS-induced Inflammatory Conditions

**Fig. 3:**
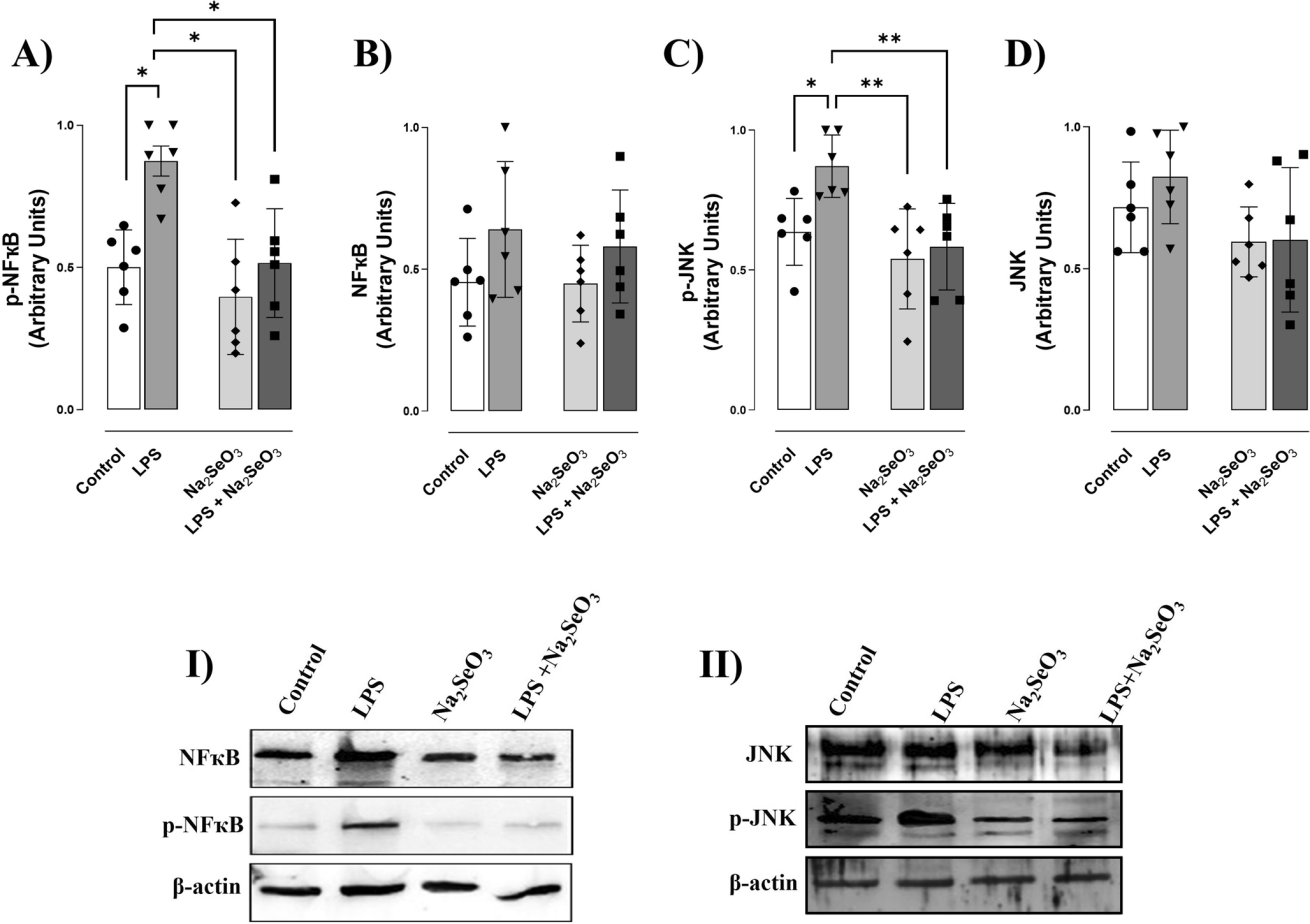
Analysis of the effects of Na_2_SeO_3_ and LPS on MSC signaling pathways. (**A**) phosphorylated NF-κB, (**B**) total NF-κB, (**C**) phosphorylated JNK and (**D**) total JNK expression by Western blot. Data are expressed as mean ± SD. (**I**) Representative membrane showing phosphorylated and total NF-κB. (**II**) Representative membrane showing phosphorylated JNK and total JNK. n = 6. (*p < 0.05; **p < 0.01 and ***p < 0.001)

To investigate the mechanisms involved in the response to Na_2_SeO_3_ treatment, the expression of proteins associated with the activation of inflammatory signaling pathways in MSCs was analyzed (Fig. 3 and **Figure ****S2**). As shown in Fig. 3A, LPS stimulation increased cellular levels of phosphorylated NFκB (p-NFκB) by approximately twofold compared with the control (basal level), with statistically significant differences relative to all other experimental groups studied, while total NFκB expression remained unchanged (Fig. 3B). Consistent with these findings, a similar pattern was observed for phosphorylated JNK expression (p-JNK) (Fig. 3C), whereas total JNK levels did not differ among the groups (Fig. 3D). However, no effective activation of these signaling pathways was detected in the presence of Na_2_SeO_3_ (Fig. 3A-D).

Additionally, to further investigate the inflammatory and oxidative signaling pathways, MSCs stimulated with LPS in the presence or absence of sodium selenite were cultured with or without N-acetylcysteine (NAC) (Figure S3). Results showed that, in NAC-treated MSCs, LPS stimulation induced NFκB phosphorylation, which remained significantly higher than in control cells (Figure S3A). In contrast, no significant differences in JNK phosphorylation were observed between control and LPS-treated MSCs cultured in the presence of NAC, although a trend toward increased JNK activation was detected following LPS stimulation. Furthermore, sodium selenite treatment reduced NFκB phosphorylation in both unstimulated and LPS-stimulated MSCs cultured with NAC. Similarly, the combination of sodium selenite and NAC resulted in lower JNK phosphorylation levels compared with the respective untreated groups (Figure S3B).

### Production of ROS by MSCs after Exposure to Na_2_SeO_3_ under LPS-induced Inflammatory Stimulus

**Fig. 4:**
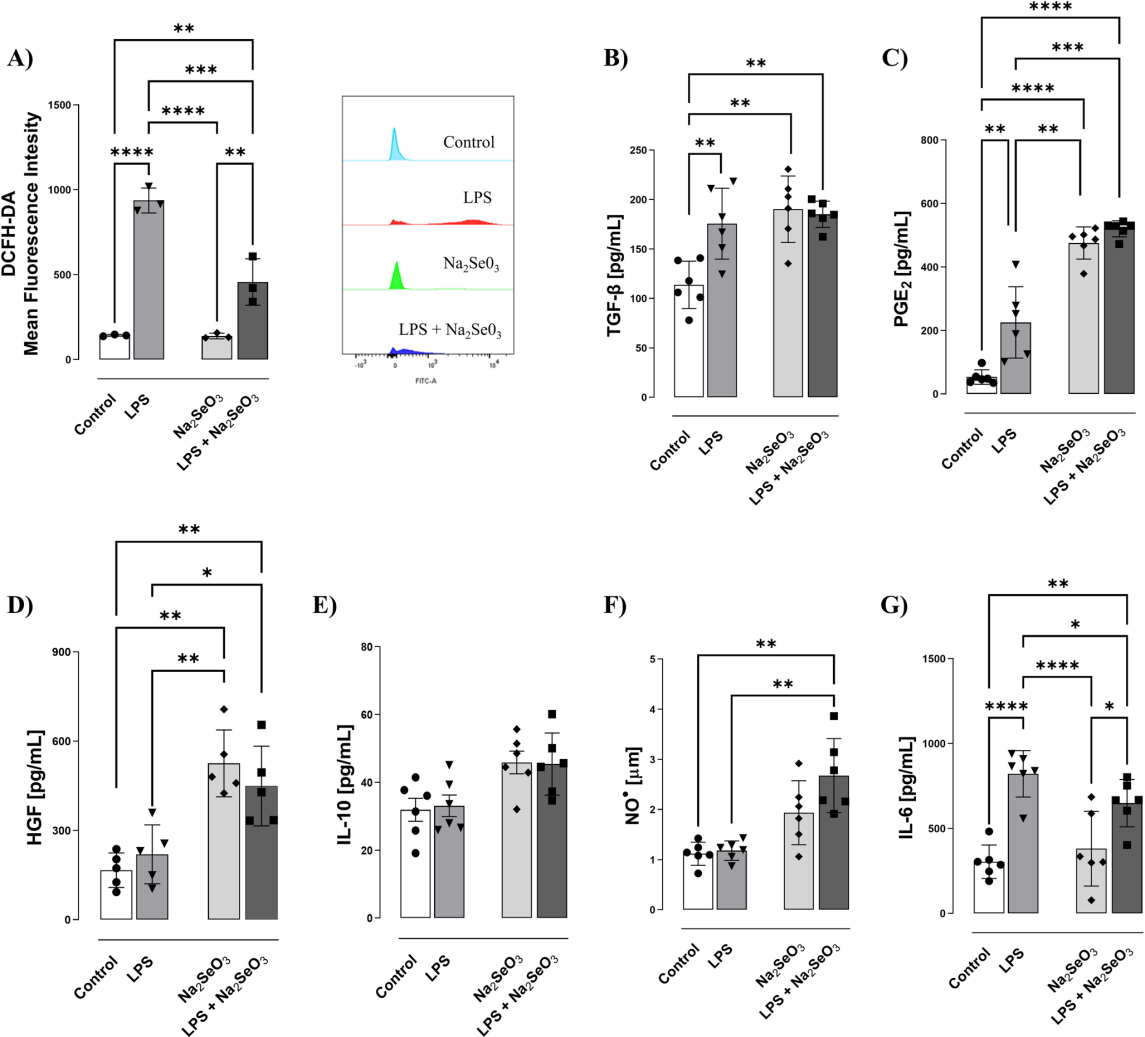
Analysis of the effects of Na_2_SeO_3_ and LPS on intracellular reactive oxygen species (ROS) and soluble factor production by MSCs. (**A**) Intracellular ROS levels were assessed by DCFH-DA and staining followed by flow cytometry. Representative histograms showing DCFH-DA fluorescence intensity are presented alongside the quantification of ROS production, expressed as mean fluorescence intensity (MFI). Data are presented as mean ± SD from three independent experiments. Production of the soluble factors (**B**) TGF-β, (**C**) PGE_2_, (**D**) HGF, (**E**) IL-10, (**F**) NO, and (**G**) IL-6 was quantified in culture supernatants. Data are presented as mean ± SD from six independent experiments. (*p < 0.05; **p < 0.01; ***p < 0.001 and ****p < 0.0001)

LPS stimulation significantly increased intracellular ROS production, as demonstrated by the marked elevation in DCFH-DA fluorescence intensity compared with the negative control group (Fig. 4A). Treatment with sodium selenite alone did not significantly alter ROS levels relative to the control. However, co-treatment with sodium selenite and LPS significantly reduced ROS production compared with the LPS group, although fluorescence levels remained higher than those observed in the control and sodium selenite-alone groups. Pretreatment with NAC markedly decreased DCFH-DA fluorescence in all experimental conditions, including LPS-stimulated cells, confirming the antioxidant activity of NAC and supporting that the observed fluorescence signal was associated with intracellular ROS generation. These findings indicate that sodium selenite partially attenuated LPS-induced oxidative stress, while NAC effectively suppressed ROS production under all tested conditions (Figure S4).

### Production of Soluble Factors by MSCs after Exposure to Na_2_SeO_3_ under LPS-Induced Inflammatory Stimulus

Considering that the immunomodulatory effects of MSCs are mainly mediated through the release of soluble factors, we evaluated the impact of Na_2_SeO_3_ on the ability of these cells to regulate the production of such molecules. According to our findings, treatment with Na_2_SeO_3_, either in the presence or absence of LPS stimulation, did not affect IL-10 production by MSCs (Fig. 4B). In contrast, exposure of MSCs to Na_2_SeO_3_, regardless of LPS stimulation, resulted in a significant increase in the production of PGE_2_ (Fig. 4C) and HGF (Fig. 4D). Regarding TGF-β production, increased levels of this cytokine were observed in all treated groups compared with the control group, with no significant differences between Na_2_SeO_3_ treatment alone and Na_2_SeO_3_ combined with LPS stimulation (Fig. 4E). However, nitric oxide (NO) production by MSCs was increased in the Na_2_SeO_3_-treated group under LPS stimulation compared with groups not exposed to Na_2_SeO_3_ (Fig. 4F). In contrast, IL-6 production (Fig. 4G) was significantly reduced in MSCs exposed exclusively to Na_2_SeO_3_, whereas LPS stimulation increased IL-6 levels.

### Effect of Na_2_SeO_3_ on MSCs Migration

**Fig. 5:**
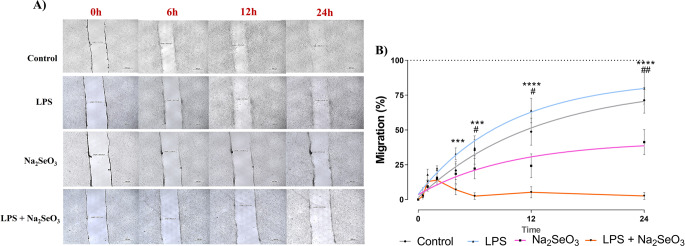
Analysis of the effects of Na_2_SeO_3_ and LPS on MSC migratory capacity. The Scratch Wound Healing Assay was performed using in vitro cell culture. (**A**) Representative micrographs were obtained at 0 h (immediately after scratching), 6 h, 12 h, and 24 h, allowing dynamic monitoring of the migratory process over time across the different treatment groups. (**B**) For quantitative analysis, a kinetic curve was generated by measuring the wound width in micrometers (µm), based on the distance between the opposing cell edges within the gap region. Data are expressed as mean ± SD. n = 3. (***p < 0.001 and ****p < 0.0001 when compared with control vs. LPS + Na_2_SeO_3_; #*p *< 0.05 and ##*p* < 0.01 when compared with control vs. Na_2_SeO_3_)

**Fig. 6:**
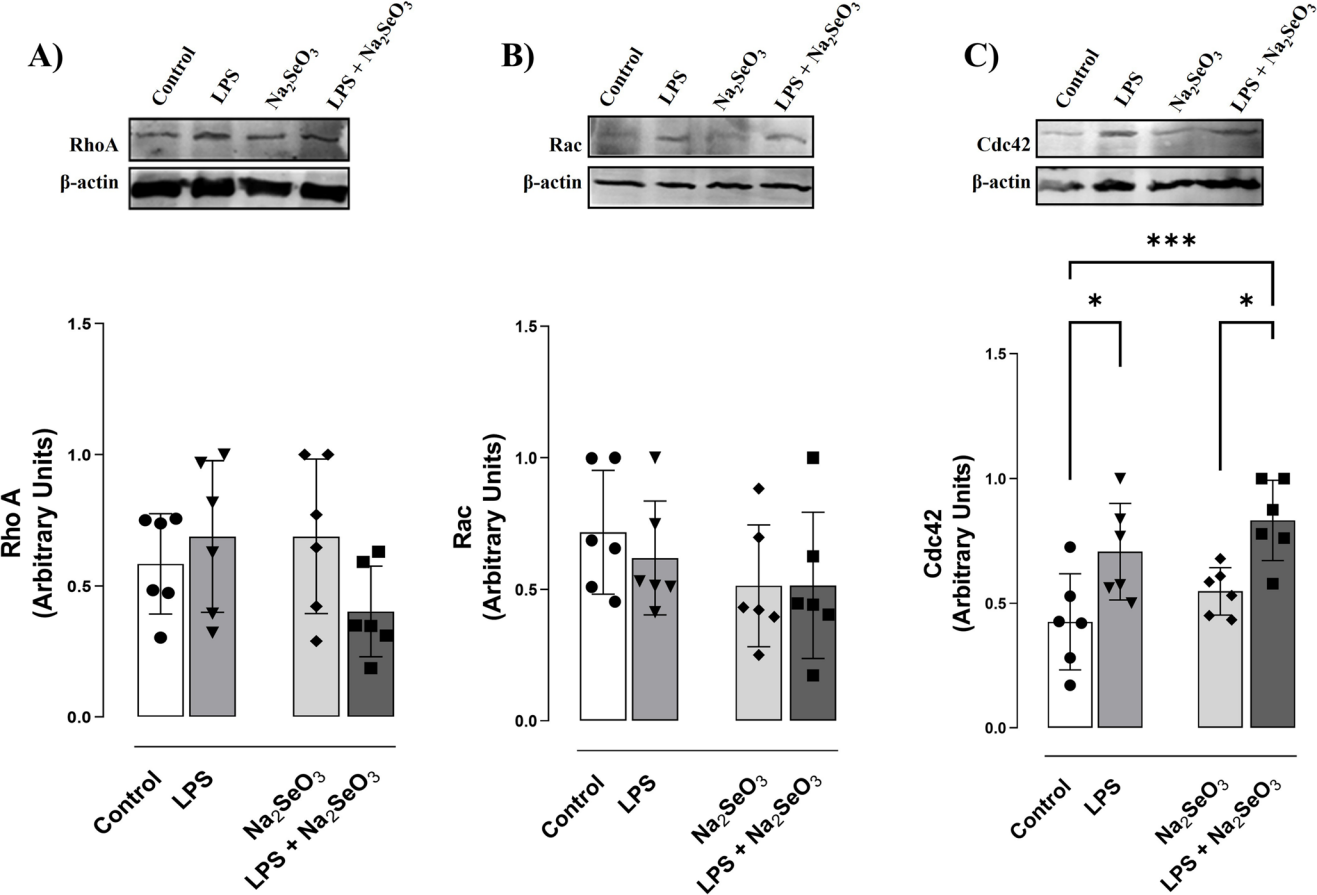
Analysis of the effects of Na_2_SeO_3_ and LPS on Rho GTPase expression in MSCs. (**A**) RhoA, (**B**) Rac1, and (**C**) Cdc42 expression by Western blot. Data are expressed as mean ± SD. n = 6. (*p < 0.05 and***p < 0.001)

Following the evaluation of soluble factor production by MSCs, we investigated whether exposure to Na_2_SeO_3_, in the presence or absence of LPS-induced inflammatory stimulation, affected the migratory capacity of these cells. As shown in Fig. 5, both the control and LPS groups exhibited progressive cell migration over the culture period. In contrast, the Na_2_SeO_3_-treated group showed significantly reduced cell migration compared with groups not treated with Na_2_SeO_3_. Similarly, the LPS + Na_2_SeO_3_–treated group did not display the expected migratory pattern, exhibiting the lowest migratory capacity among all evaluated groups. However, no differences among groups were observed when total RhoA and Rac protein levels were measured (Fig. 6A and B, respectively; Figure S2). However, the quantification of Cdc42 expression revealed that groups treated with LPS, regardless of Na_2_SeO_3_ supplementation, exhibited higher Cdc42 protein levels compared with their respective control groups (Fig. 6C; Figure S2).

### Na_2_SeO_3_ Treatment Combined with MSCs-Conditioned Medium Suppresses Pro-inflammatory Cytokine Production and NFκB Activation in Macrophages

**Fig. 7:**
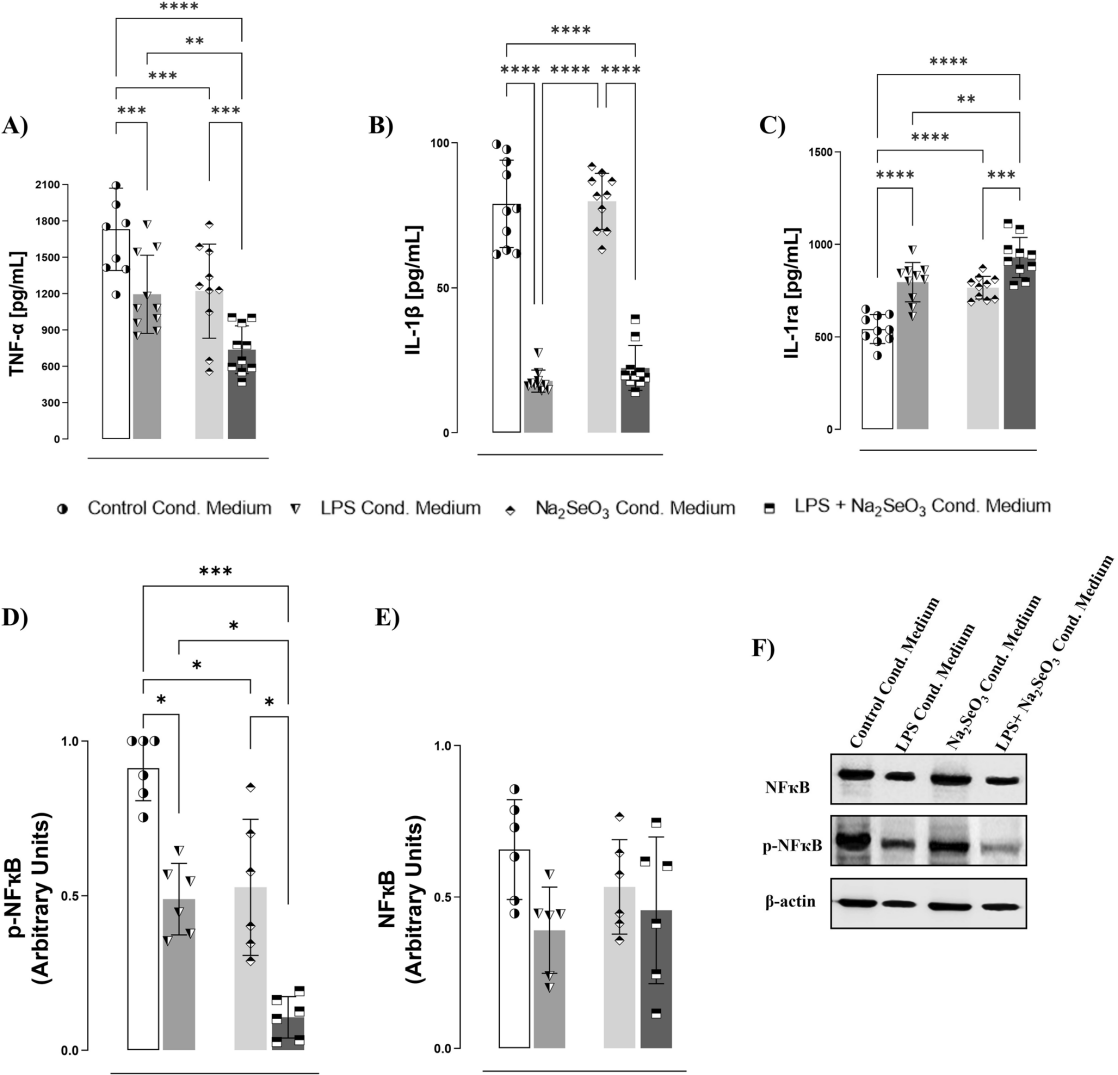
Effects of MSC-conditioned medium on mono-macrophage cells. Mono-macrophage cells were incubated for 24 h with MSC-conditioned medium, in the presence of Na_2_SeO_3_ and LPS stimulation. The production of (**A**) TNF-α,(**B**) IL-1β, and (**C**) IL-1ra by macrophages was measured (n = 10). Protein expression levels of (**D**) phosphorylated NF-κB and (**E**) total NF-κB were also quantified by Western blot. (F) Representative membrane showing phosphorylated and total NF-κB expression (n = 6). Data are expressed as mean ± SD. (*p < 0.05; **p < 0.01; ***p < 0.001 and ****p < 0.0001)

To evaluate whether factors secreted by MSCs can alter the inflammatory profile of RAW 264.7 macrophages, the production of cytokines associated with pro- and anti-inflammatory responses after LPS stimulus was assessed, including TNF-α, IL-1β, and IL-1ra (Fig. 7; cytokine levels were also evaluated in the absence of LPS stimulation, Figure S5). The results showed that groups treated in the absence of MSCs-conditioned medium exhibited increased release of TNF-α (Fig. 7A) and IL-1β (Fig. 7B), when stimulated with LPS. In contrast, groups cultured with conditioned medium exhibited reduced production of these pro-inflammatory cytokines. This inhibitory effect was especially pronounced for TNF-α production in cells treated with conditioned medium derived from the LPS + Na_2_SeO_3_–treated group (Fig. 7A and B) without affecting macrophage viability (Figure S5). Furthermore, IL-1ra production (Fig. 7C) was increased in groups cultured with conditioned medium, particularly when derived from MSCs treated with LPS + Na_2_SeO_3_, whereas lower levels were observed in groups cultured without conditioned medium, supporting the establishment of an anti-inflammatory cytokine profile. Based on these findings, NFκB expression in macrophages was also evaluated (Fig. 7D–F; Figure S2), revealing reduced phosphorylation levels (Fig. 7D), especially in macrophages exposed to conditioned medium from LPS + Na_2_SeO_3_–treated MSCs.

## Discussion

The present study investigated whether sodium selenite-mediated preconditioning functionally modulates MSC behavior under inflammatory conditions and how these alterations influence macrophage responses through paracrine mechanisms. Rather than inducing overt cytotoxicity, Na_2_SeO_3_ preconditioning primarily reshaped cellular signaling and immunomodulatory properties of MSCs, revealing a context-dependent regulatory effect strongly influenced by inflammatory stimulation.

Selenium is recognized for its bimodal biological activity, acting either as an antioxidant or a pro-oxidant depending on concentration, exposure time, and cellular context [16, 17]. The concentration employed here was intentionally selected within the upper range previously reported to induce cellular stress responses [27, 28] to investigate how high selenium exposure influences MSCs regulatory capacity rather than systemic selenium physiology. Importantly, the absence of marked metabolic impairment indicates that high Na_2_SeO_3_ exposure does not necessarily translate into immediate loss of cellular viability, demonstrating that MSCs remain highly resistant even under elevated selenium concentrations. These findings reinforce the concept that controlled exposure to stress-associated stimuli may induce adaptive functional responses in MSCs rather than simple cytotoxic injury, supporting the rationale of selenium-mediated preconditioning strategies.

In this way, the literature has already reported that MSCs display remarkable resistance to oxidative stress and inflammatory injury, largely attributed to constitutive antioxidant defenses, metabolic plasticity, and activation of pro-survival signaling pathways that limit apoptosis under hostile microenvironmental conditions [29–31]. A key finding of this study is that Na_2_SeO_3_ alone did not markedly induce apoptosis; however, its combination with LPS significantly enhanced apoptotic signaling. Despite this increase, overall cellular viability remained above 90%. This interaction suggests that selenium does not act solely as a toxic agent but may functionally modulate MSC responses by influencing signaling events initiated by inflammatory stimuli [17, 32, 33]. Although the concentration employed in this study is relatively high, previous research evaluating elevated doses of Na_2_SeO_3_ has demonstrated an absence of DNA damage, suggesting a lack of genotoxicity under these specific conditions [34, 35].

Mechanistically, these functional changes were accompanied by attenuation of NFκB and JNK signaling pathways. Both pathways represent central regulators of inflammatory activation and cellular stress responses [25, 36]. Their inhibition suggests that Na_2_SeO_3_ may be associated with negative regulatory mechanisms capable of restraining excessive endotoxin-driven signaling. Corroborating these findings, a study demonstrated that Na_2_SeO_3_ was able to significantly inhibit LPS-stimulated activation of the NFκB and MAPK pathways, in addition to exerting anti-inflammatory effects in mastitis models [37]. Importantly, suppression of these pathways under pro-oxidant conditions may initially appear paradoxical; however, inhibition of inflammatory cascades has been widely described as a protective mechanism preventing chronic immune activation [37, 38].

To determine whether the effects of Na_2_SeO_3_ on NFκB and JNK signaling were associated with redox regulation, MSCs were treated with N-acetylcysteine (NAC), a well-established antioxidant and glutathione precursor that scavenges reactive oxygen species (ROS) and restores intracellular redox balance [39]. As expected, LPS stimulation significantly increased ROS production in MSCs, whereas Na_2_SeO_3_ partially attenuated this response. Pretreatment with NAC markedly reduced ROS levels under all experimental conditions, confirming the contribution of oxidative stress to LPS-induced cellular activation (Figure S4). Despite effective ROS suppression, NFκB phosphorylation remained significantly elevated in LPS-stimulated MSCs compared with control cells, suggesting that NFκB activation is not exclusively dependent on ROS generation (Figure S3A). In contrast, LPS-induced JNK activation was attenuated in the presence of NAC and no longer differed significantly from control cells, although a trend toward increased JNK phosphorylation persisted following LPS stimulation. Furthermore, combined treatment with NAC and Na_2_SeO_3_ resulted in marked suppression of JNK phosphorylation (Figure S3B). Moreover, the ability of Na_2_SeO_3_ to suppress NFκB signaling even under antioxidant conditions suggests that ROS scavenging alone does not fully explain its immunomodulatory effects.

Additionally, the observed reduction in migratory capacity may reflect the acquisition of an anti-inflammatory MSCs phenotype, signaling a shift toward enhanced immunomodulatory function rather than compromised viability [7, 40]. While total RhoA and Rac protein expression, which act as key regulators of cytoskeletal organization and cell behavior [41], remained unchanged, this may be attributed to the measurement of total protein rather than their active GTP-bound isoforms, which more directly govern migratory signaling [42]. In contrast, Cdc42 expression was upregulated following LPS stimulation. Given that Cdc42 participates not only in cell migration but also in cell cycle progression, its upregulation, alongside the observed S/G2/M accumulation, may indicate a compensatory signaling mechanism or a stress-induced attempt to maintain cytoskeletal integrity during these specific phases of the cell cycle [41, 42].

In addition, Na_2_SeO_3_-mediated preconditioning markedly influenced the MSC secretory profile. Increased production of regulatory mediators such as TGF-β, NO and HGF, together with suppression of inflammatory signaling, is consistent with the acquisition of an anti-inflammatory phenotype [43, 44]. Interestingly, IL-10 production remained unchanged, suggesting that selenium-mediated immunomodulation does not necessarily depend on classical anti-inflammatory cytokines [45]. Instead, MSC function appears to rely predominantly on coordinated networks of soluble mediators.

Among these mediators, PGE_2_ may represent an important contributor to the immunomodulatory effects observed following Na_2_SeO_3_ preconditioning [7, 9]. Although PGE_2_ was not the primary mechanistic focus of this study, its increased production in Na_2_SeO_3_-treated MSCs is consistent with previous reports demonstrating selenium-induced enhancement of PGE_2_ synthesis [46, 47]. PGE_2_ signaling has been associated with macrophage polarization through EP2 and EP4 receptor activation, promoting cyclic AMP–dependent anti-inflammatory responses [47]. The integrated increase in regulatory cytokines and inhibition of NFκB signaling observed in this study aligns with this mechanism.

The functional relevance of Na_2_SeO_3_-mediated MSC preconditioning became evident when conditioned media were applied to macrophages. Exposure of macrophages to MSC-derived soluble factors significantly reduced TNF-α and IL-1β production, while increasing IL-1 receptor antagonist (IL-1ra) levels. Rather than simply suppressing inflammation, this pattern reflects active immune reprogramming [48, 49]. Increased IL-1ra competes with IL-1β for receptor binding, attenuating downstream signaling and favoring resolution of inflammation [48]. Such receptor-level antagonism represents an efficient mechanism through which MSCs regulate immune activation without eliminating immune competence [7, 48].

Taken together, these findings support a model in which high-dose Na_2_SeO_3_-mediated preconditioning is associated with suppression of pro-inflammatory pathways, reduced migratory activity, and enhanced immunomodulatory secretory function of MSCs under inflammatory conditions. Under inflammatory conditions, these phenotypic alterations translate into the effective regulation of macrophage responses via paracrine signaling. From a broader perspective, these results highlight the remarkable plasticity of MSCs in response to environmental stressors.

Nevertheless, significant questions remain. Data concerning the impact of high-concentration selenium on MSC immunobiology are still emerging, and the precise molecular links between selenium-mediated modulation and long-term functional outcomes warrant further investigation. Although C3H10T1/2 cells constitute a widely used and well-characterized mesenchymal stromal model, future studies using primary MSCs and in vivo experimental systems will be important to further validate the translational relevance of these findings. In addition, future investigations should characterize dose–response relationships, temporal dynamics, and redox-associated mechanisms involved in selenium-mediated MSC functional modulation.

Furthermore, although the macrophage experiments strongly suggest that sodium selenite-mediated preconditioning alters the MSC secretory profile and paracrine immunomodulatory activity, additional studies will be important to better characterize selenium bioavailability in conditioned media and to further define the relative contribution of residual selenium versus MSC-derived soluble mediators in the regulation of macrophage inflammatory responses.

Importantly, direct exposure of RAW 264.7 macrophages to 20 µM sodium selenite under our experimental conditions resulted in marked cytotoxicity, whereas macrophages exposed to conditioned media derived from sodium selenite-preconditioned MSCs remained viable (**Figure ****S4****D**). In addition, preliminary observations obtained during the course of this study suggested that MSCs actively uptake and/or metabolize sodium selenite during the preconditioning period, potentially reducing selenium bioavailability in the conditioned medium after 24 h of exposure. Although these observations were not mechanistically investigated and remain beyond the scope of the present study, they further support the interpretation that macrophages exposed to MSC-conditioned media were not subjected to the same selenium conditions originally applied during MSC preconditioning. These findings indicate that the observed immunomodulatory effects were predominantly associated with MSC-derived paracrine modulation induced by selenium preconditioning rather than with direct selenium-mediated effects.

In conclusion, Na_2_SeO_3_-mediated preconditioning functionally reprograms MSCs by enhancing their immunomodulatory potency and modulating intracellular signaling pathways. This selenium-dependent priming was associated with the development of a regulatory secretory profile linked to reduced pro-inflammatory cytokine production and enhanced anti-inflammatory macrophage responses.

## Supplementary Information

Below is the link to the electronic supplementary material.

Supplementary Material 1 (PDF 451 KB)

Supplementary Material 2 (PDF 581 KB)

Supplementary Material 3 (PDF 464 KB)

Supplementary Material 4 (PDF 345 KB)

Supplementary Material 5 (PDF 440 KB)

## Data Availability

No datasets were generated or analysed during the current study.
