# Supplementary material for: Sodium Selenite Preconditioning Reprograms Mesenchymal Stem Cell Behavior Under Inflammatory Stress to Enhance Anti-Inflammatory Macrophage Responses
Source: Biol Trace Elem Res. 2026 Jul 3;204(9):7358–72. doi: 10.1007/s12011-026-05192-5 (PMC13437566; doi:10.1007/s12011-026-05192-5)
Supplement: Supplementary file 1 [file 12011_2026_5192_MOESM1_ESM.pdf]

Figure S1.

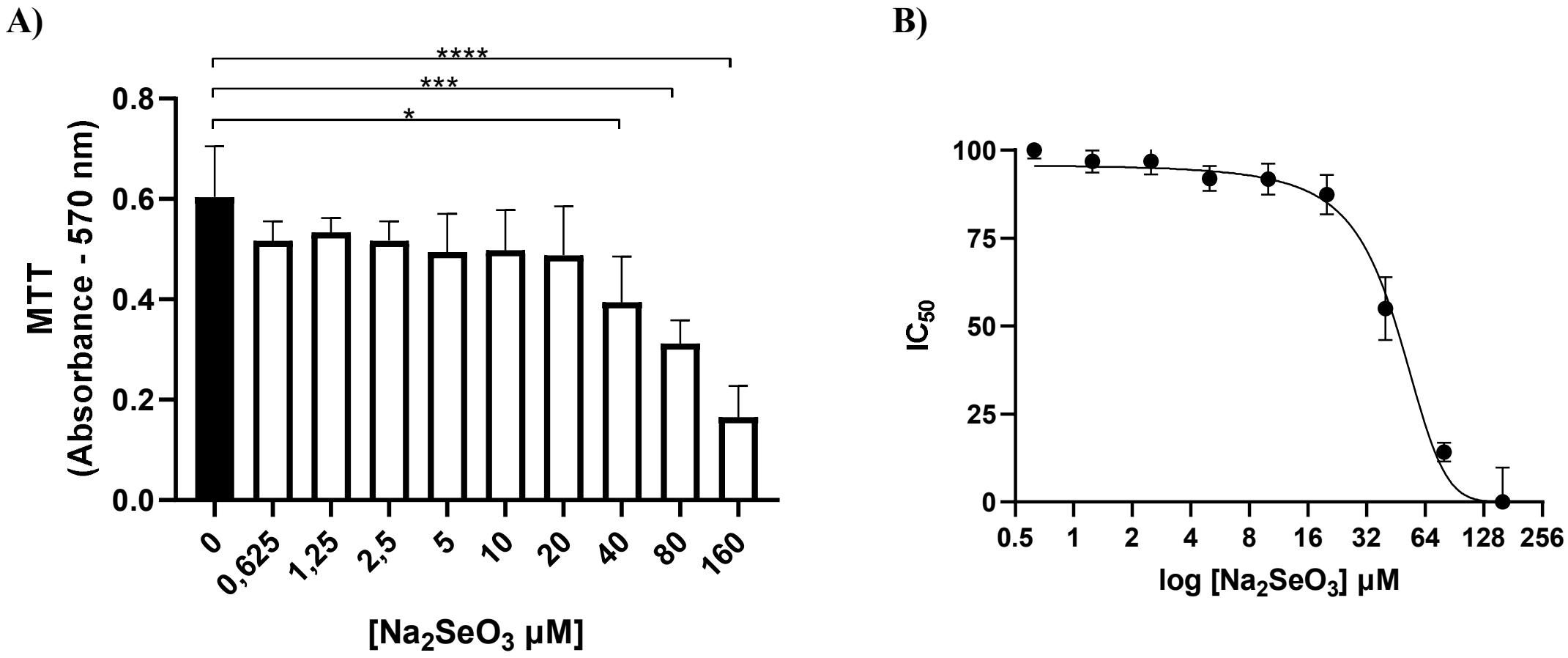

Figure S1. Effect of Na_2SeO_3 treatment on MSCs after 24 hours. (**A**) MSCs were exposed to different concentrations of Na_2SeO_3 (x-axis) and subsequently incubated with MTT. Optical density measured at 570 nm (y-axis) positively correlated with cellular metabolic activity. Data are presented as mean ± SD from the MTT assay performed with ten different concentrations of Na_2SeO_3 (**B)** Mean inhibitory concentration (IC50=45,47µM) calculated from the ten Na_2SeO_3 concentrations tested. $n = 6$. (**p < 0.05; *** p < 0.001* and **** *p < 0.0001*).
