# Supplementary material for: Sodium Selenite Preconditioning Reprograms Mesenchymal Stem Cell Behavior Under Inflammatory Stress to Enhance Anti-Inflammatory Macrophage Responses
Source: Biol Trace Elem Res. 2026 Jul 3;204(9):7358–72. doi: 10.1007/s12011-026-05192-5 (PMC13437566; doi:10.1007/s12011-026-05192-5)

Figure 3A. Full, uncropped Western blot membranes for phosphorylated and total NFκB, corresponding to the data presented in figure 3.

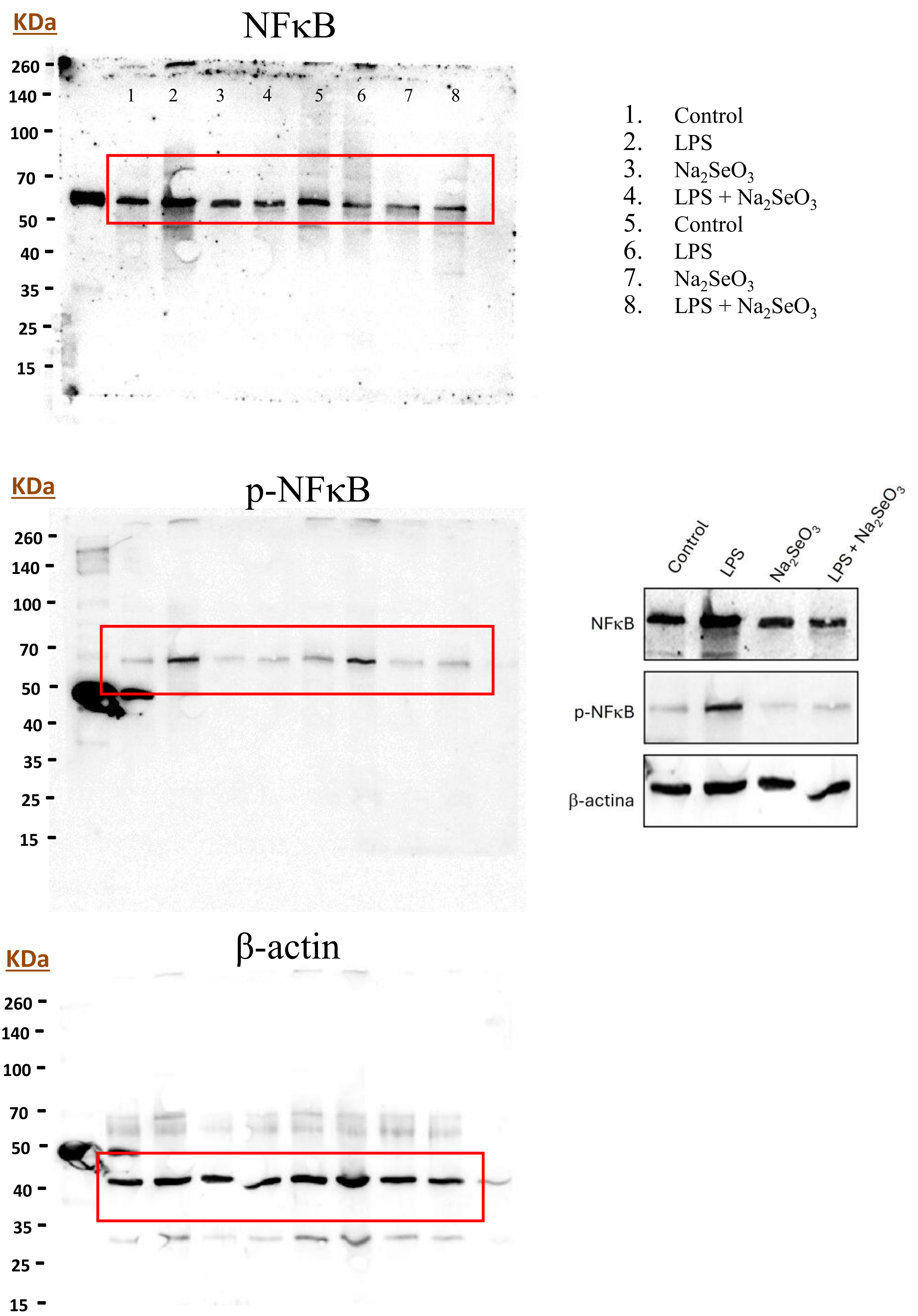

Figure 3B. Full, uncropped Western blot membranes for phosphorylated and total JNK, corresponding to the data presented in figure 3.

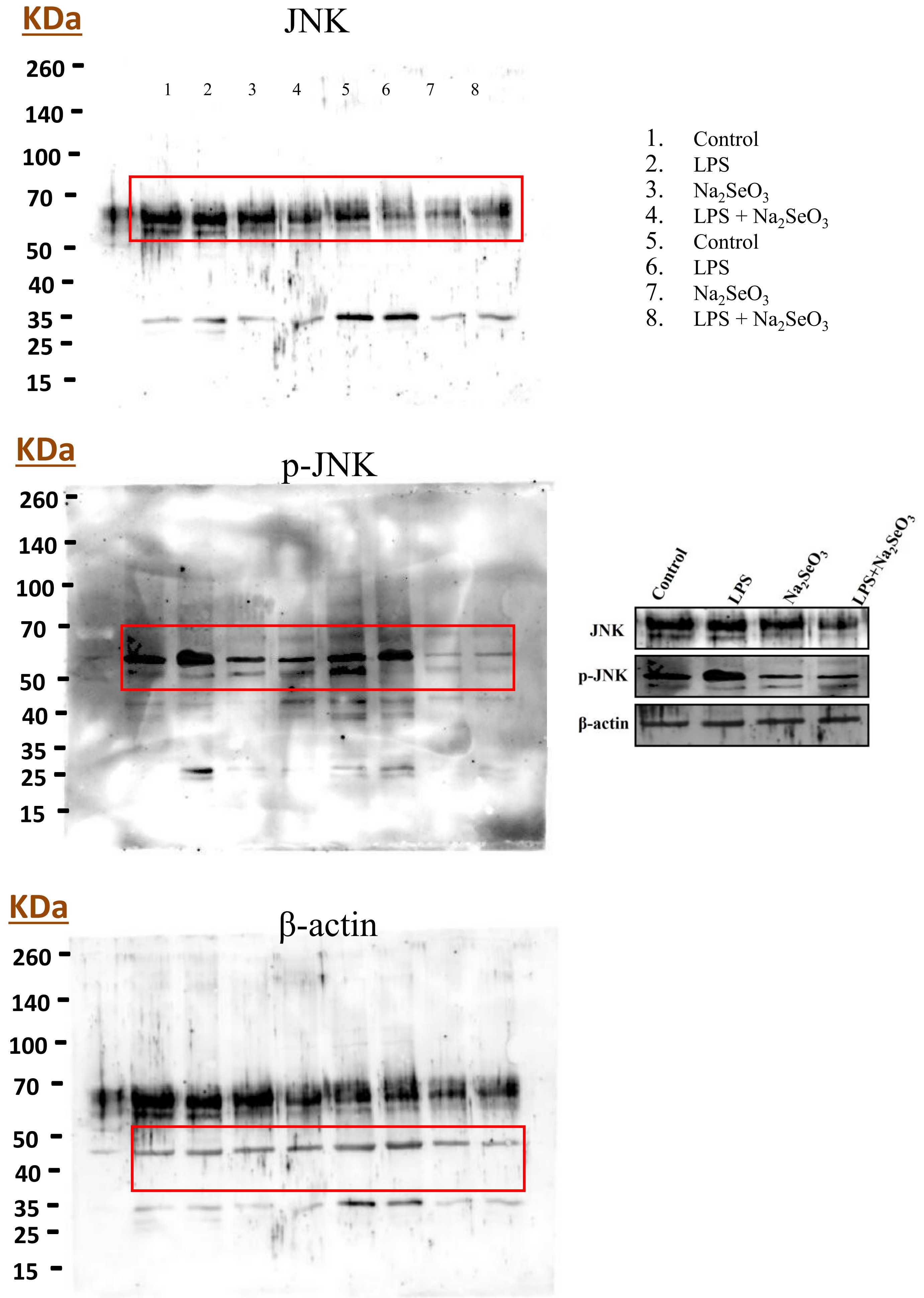

Figure 6A. Full, uncropped Western blot membranes for phosphorylated and total RhoA, corresponding to the data presented in figure 6.

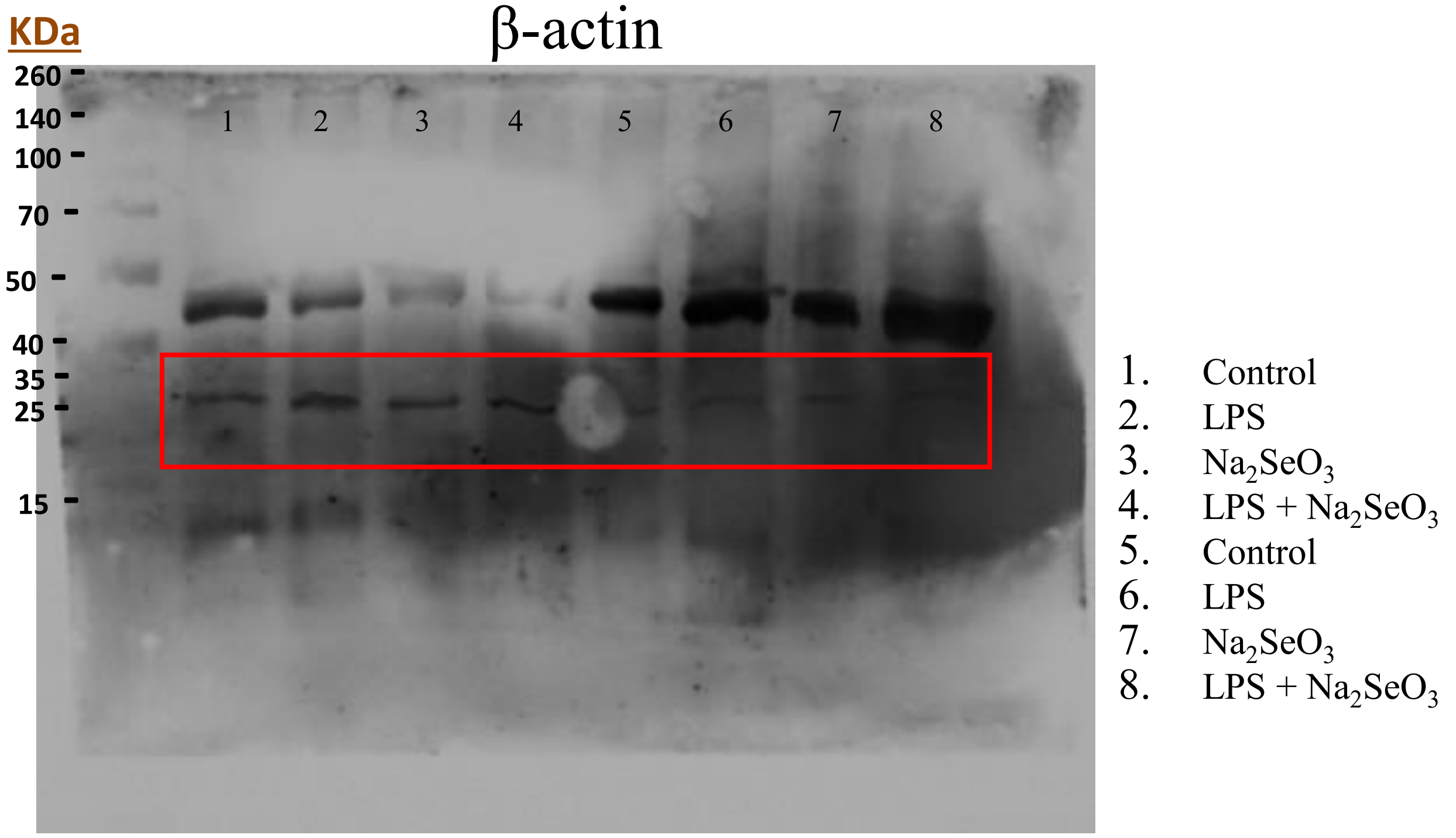

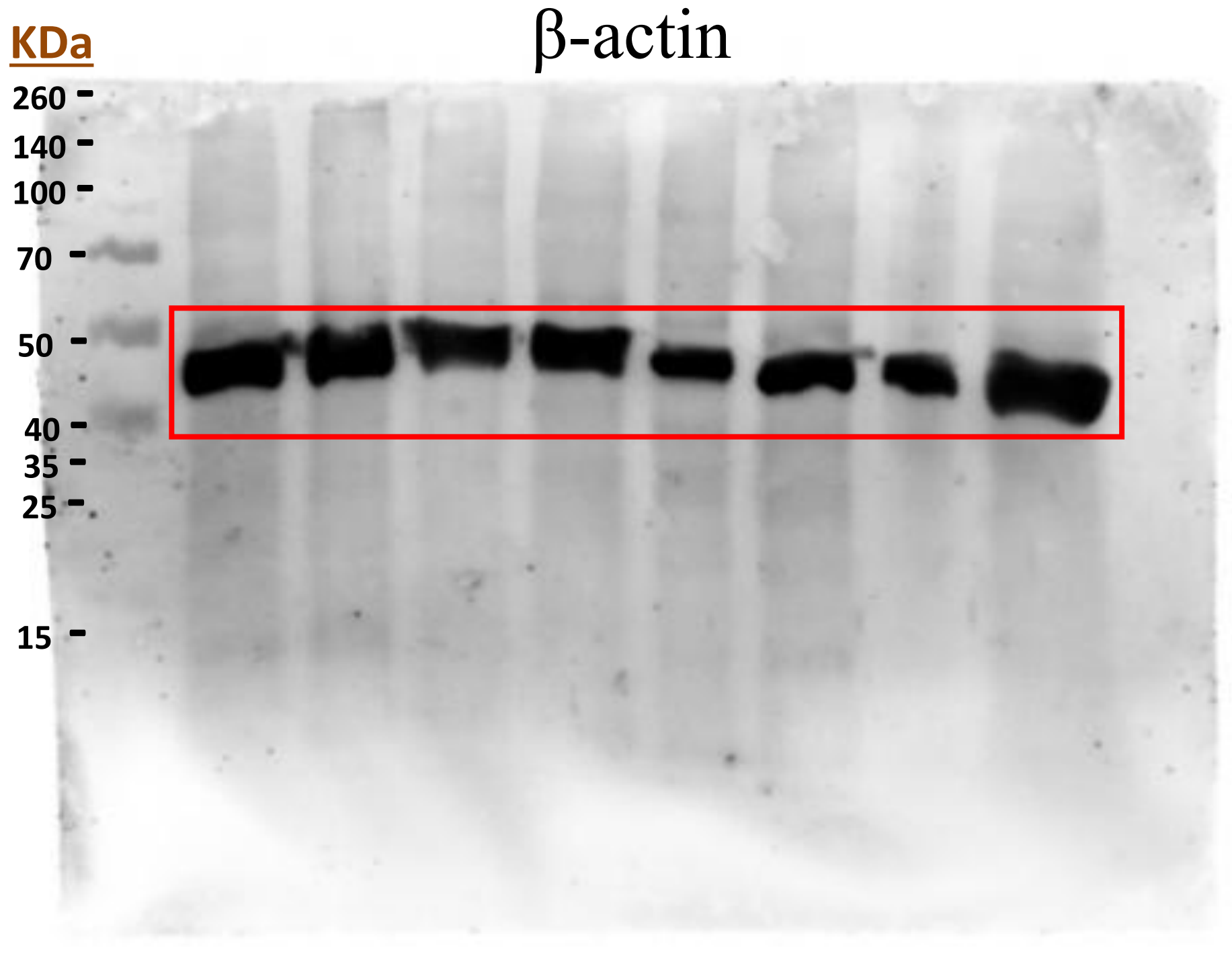

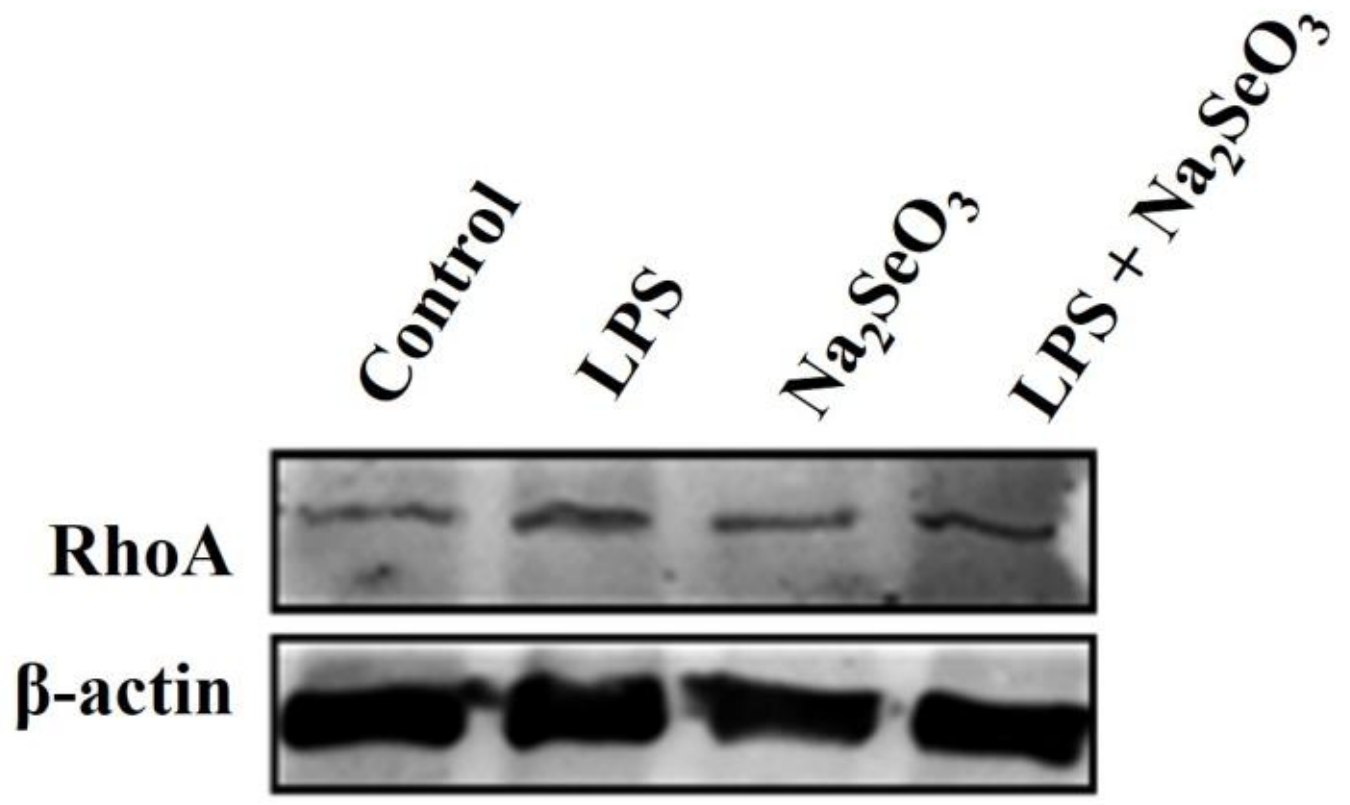

Figure 6B. Full, uncropped Western blot membranes for phosphorylated and total Rac, corresponding to the data presented in figure 6.

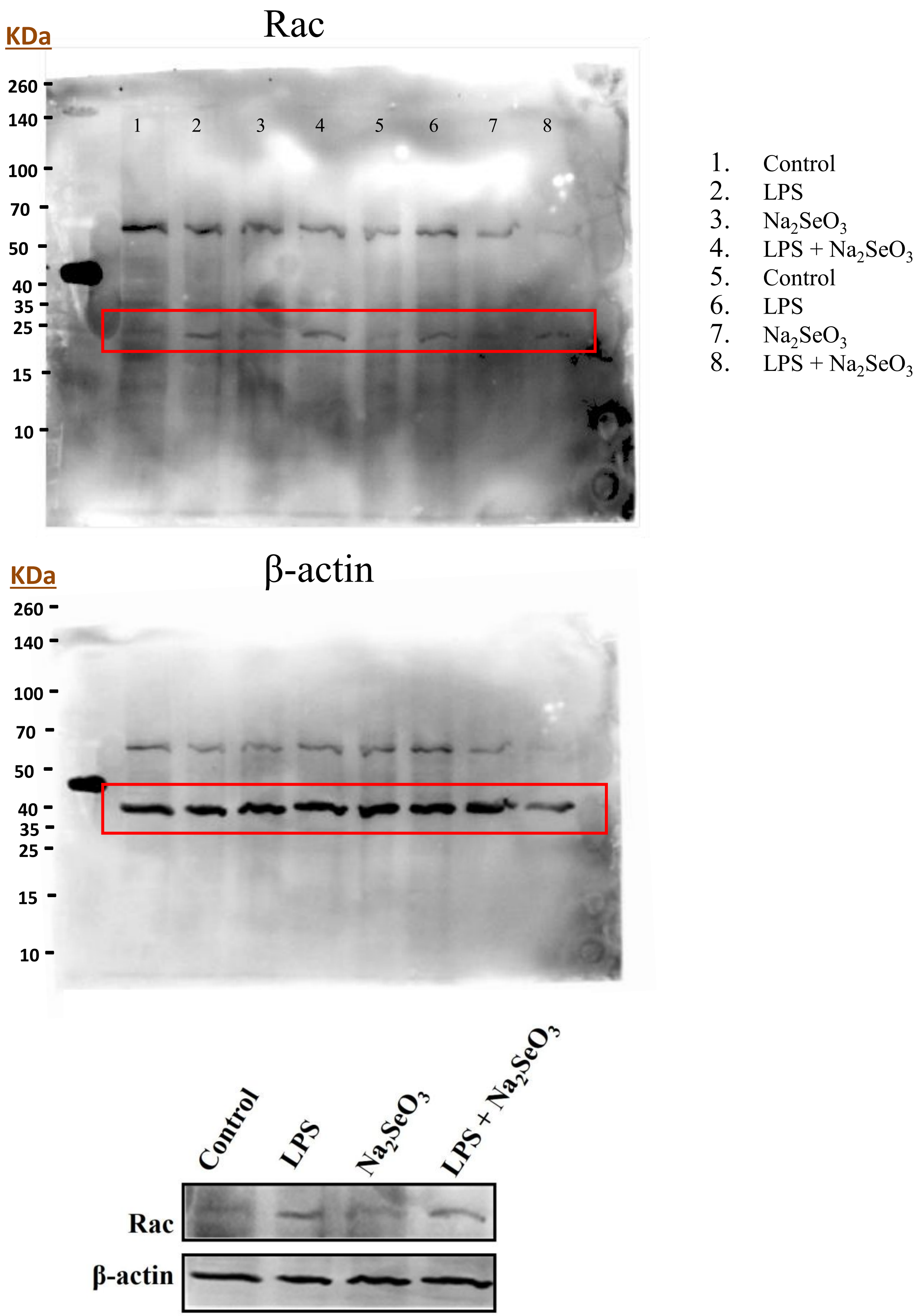

Figure 6C. Full, uncropped Western blot membranes for phosphorylated and total Cdc42, corresponding to the data presented in figure 6.

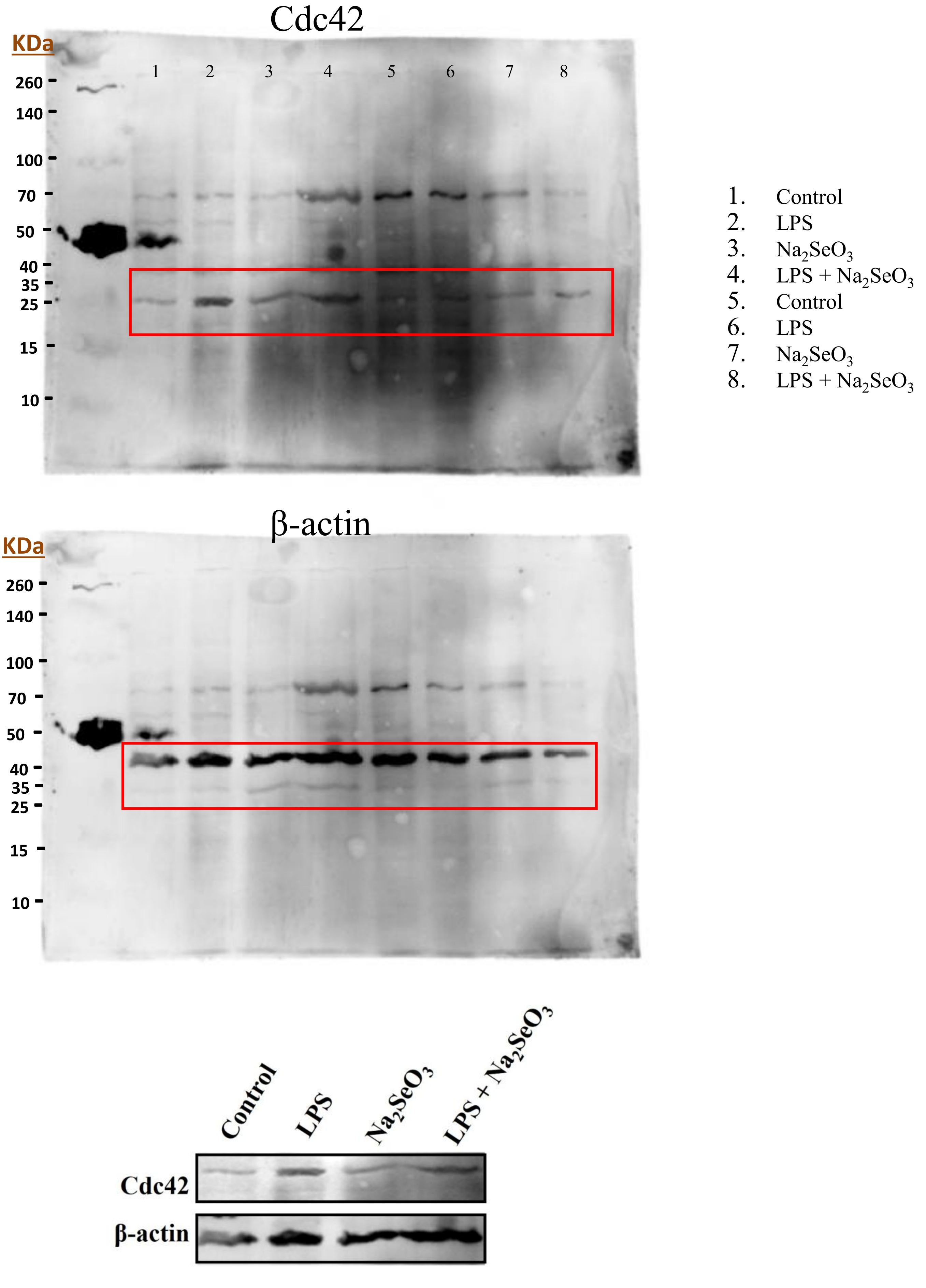

Figure 7F. Full, uncropped Western blot membranes for phosphorylated and total NFκB, corresponding to the data presented in figure 7.

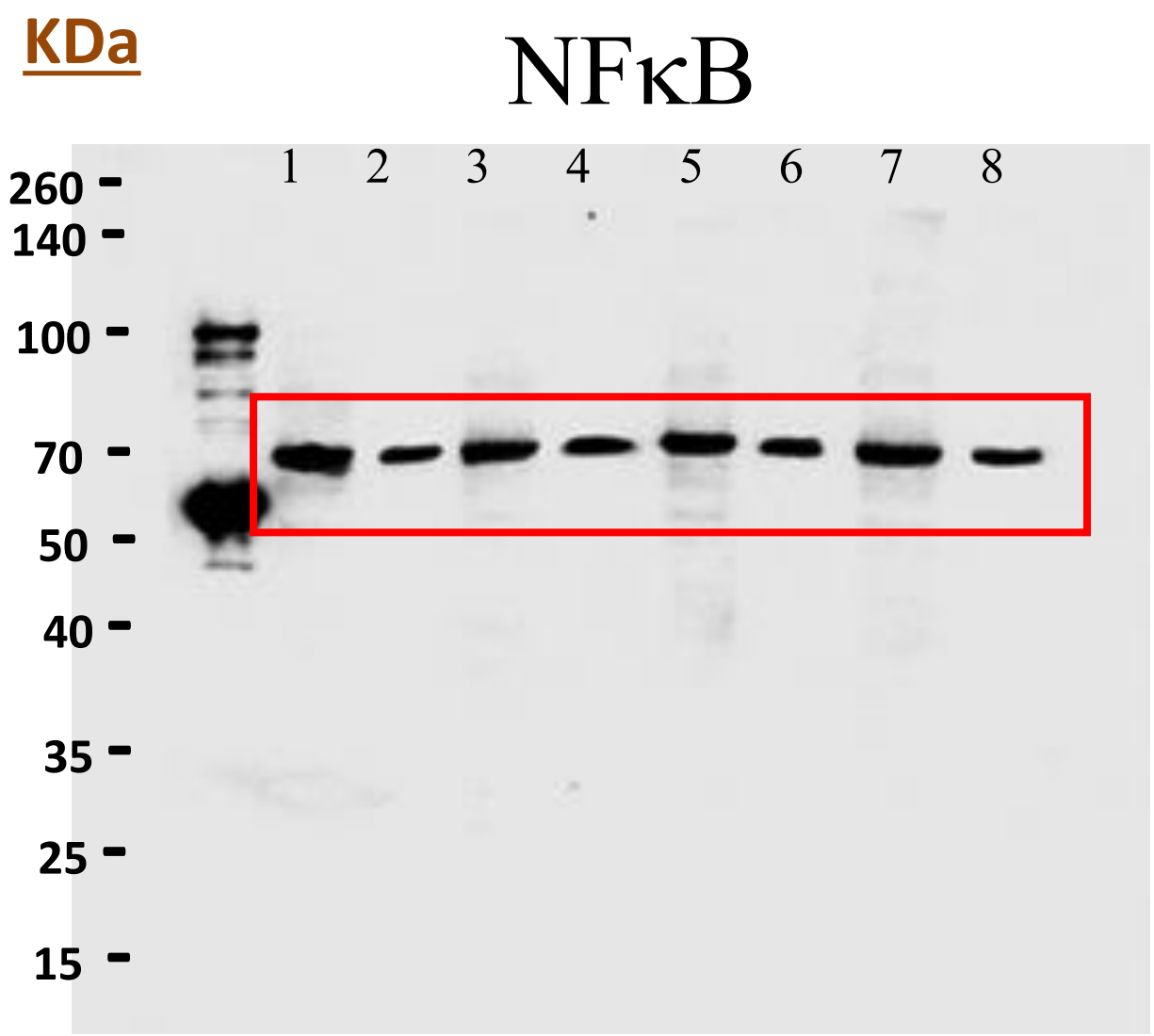

1. Control
2. Cond. Medium
3. Na_2SeO_3
4. Na_2SeO_3 /Cond. Medium
5. Control
6. Cond. Medium
7. Na_2SeO_3
8. Na_2SeO_3 /Cond. Medium

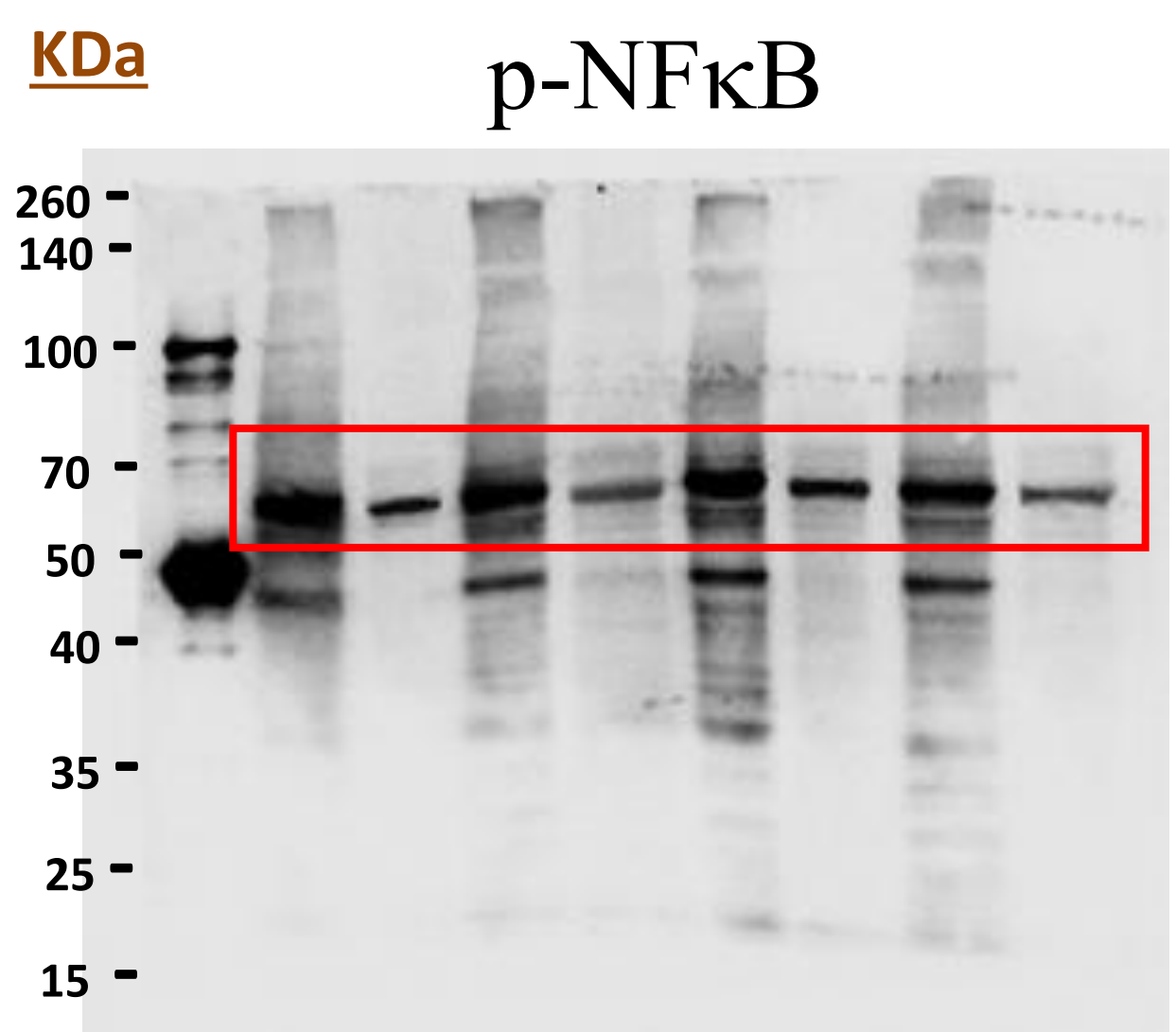

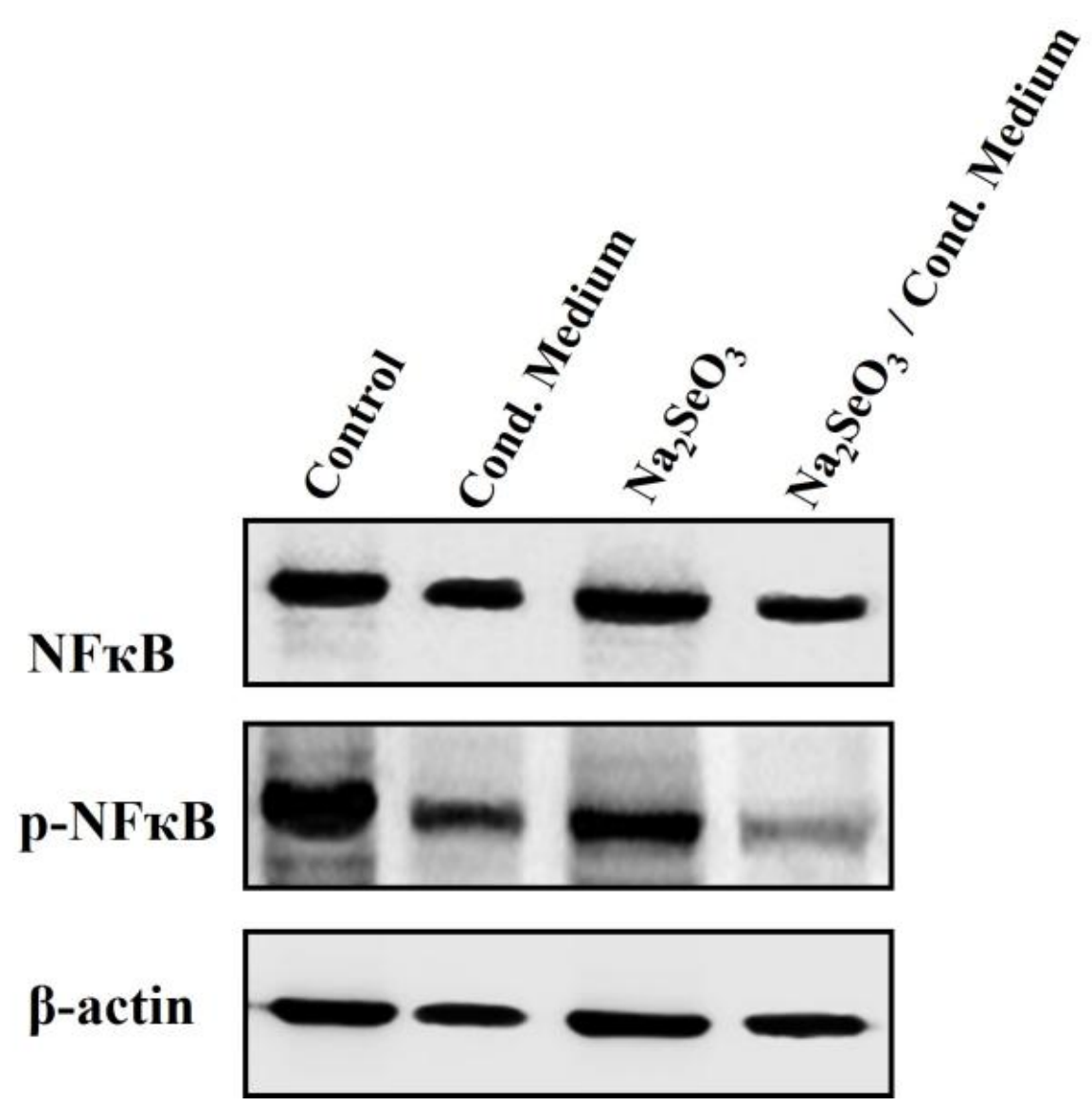

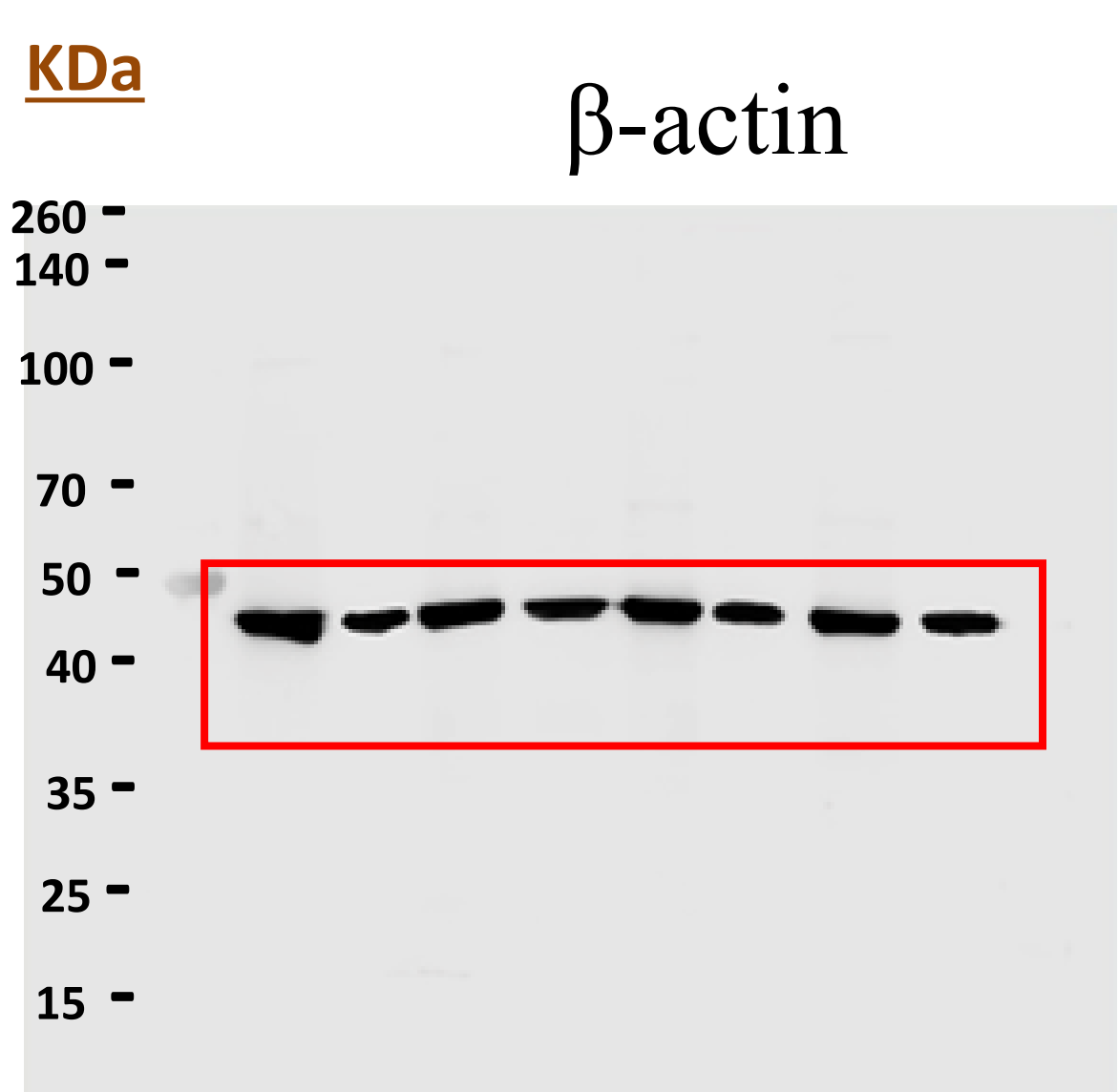

Supplement: Supplementary file 2 [file 12011_2026_5192_MOESM2_ESM.pdf]
