# Supplementary material for: Sodium Selenite Preconditioning Reprograms Mesenchymal Stem Cell Behavior Under Inflammatory Stress to Enhance Anti-Inflammatory Macrophage Responses
Source: Biol Trace Elem Res. 2026 Jul 3;204(9):7358–72. doi: 10.1007/s12011-026-05192-5 (PMC13437566; doi:10.1007/s12011-026-05192-5)
Supplement: Supplementary file 3 [file 12011_2026_5192_MOESM3_ESM.pdf]

Figure S3.

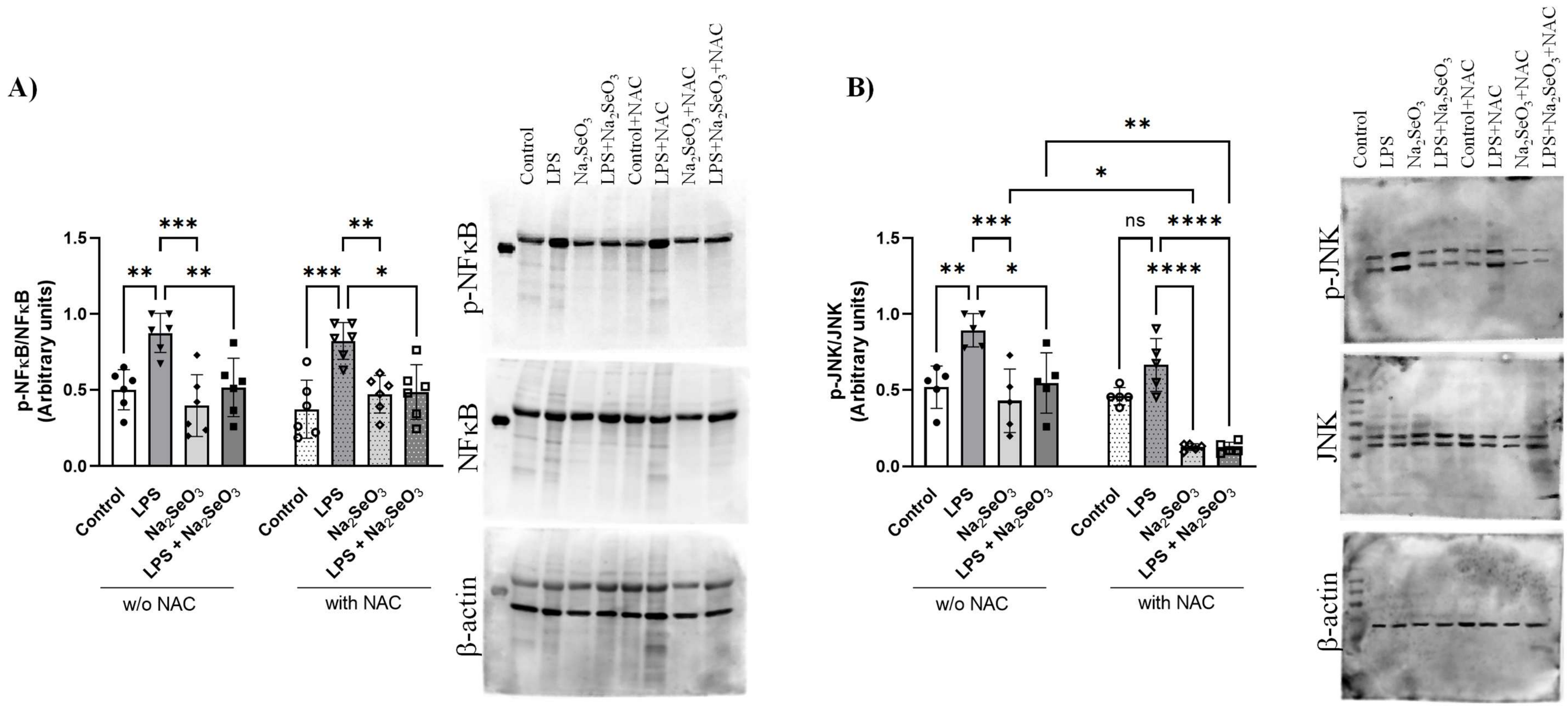

Figure S3. Effect of Na_2SeO_3 and NAC on NFκB and JNK phosphorylation in MSCs under inflammatory conditions. (**A**) MSCs were treated with LPS, Na_2SeO_3, NAC, or their respective combinations for 24 h. Protein expression of phosphorylated NF-κB (p-NF-κB) and total NF-κB was evaluated by Western blotting. Densitometric analysis of p-NF-κB levels normalized to total NF-κB is shown in the bar graphs (left panels), while representative immunoblots for p-NF-κB, NF-κB, and β-actin are presented on the right. Data are presented as mean ± SD from six independent experiments. (**B**) Protein expression of phosphorylated JNK (p-JNK) and total JNK was analyzed by Western blotting under the same experimental conditions. Densitometric quantification of p-JNK normalized to total JNK is shown in the bar graphs (left panels), and representative immunoblots for p-JNK, JNK, and β-actin are displayed on the right. Data are presented as mean ± SD from five independent experiments. (*$p < 0.05$; ** $p < 0.01$; *** $p < 0.001$ and **** $p < 0.0001$).
