# Supplementary material for: Sodium Selenite Preconditioning Reprograms Mesenchymal Stem Cell Behavior Under Inflammatory Stress to Enhance Anti-Inflammatory Macrophage Responses
Source: Biol Trace Elem Res. 2026 Jul 3;204(9):7358–72. doi: 10.1007/s12011-026-05192-5 (PMC13437566; doi:10.1007/s12011-026-05192-5)
Supplement: Supplementary file 4 [file 12011_2026_5192_MOESM4_ESM.pdf]

Figure S4.

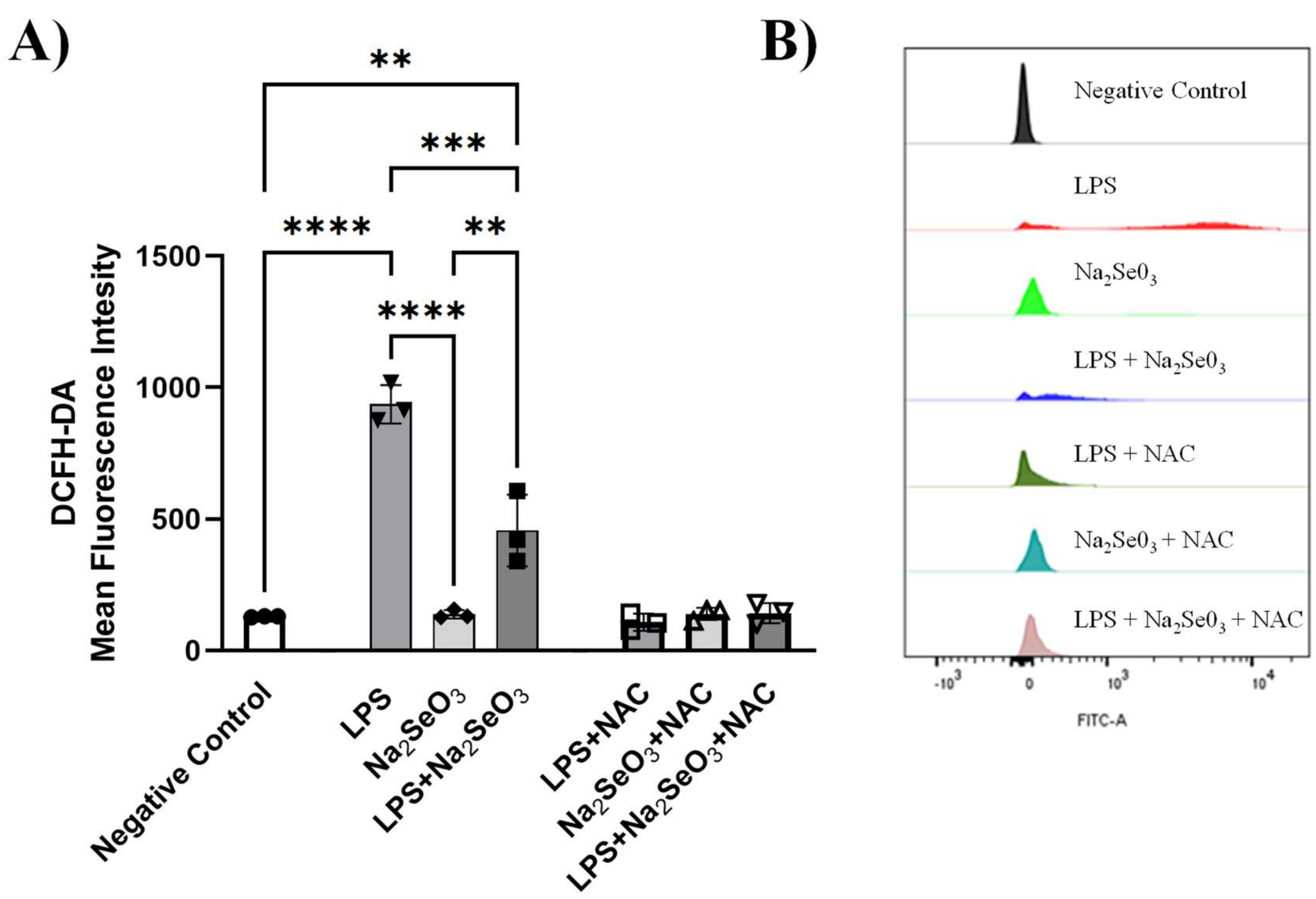

Figure S4. Effect of Na_2SeO_3 and NAC on intracellular ROS production in MSCs after 24 hours. (**A**) MSCs were treated with LPS, Na_2SeO_3, NAC, or their respective combinations for 24 h, and intracellular reactive oxygen species (ROS) levels were assessed by DCFH-DA staining followed by flow cytometry. Mean fluorescence intensity (MFI) of DCFH-DA (y-axis) was used as an indicator of intracellular ROS accumulation. Data are presented as mean ± SD. (**B**) Representative flow cytometry histograms showing DCFH-DA fluorescence intensity in MSCs under the indicated treatment conditions. n = 3. (**$p < 0.01$; *** $p < 0.001$ and **** $p < 0.0001$).
