# Supplementary material for: Sodium Selenite Preconditioning Reprograms Mesenchymal Stem Cell Behavior Under Inflammatory Stress to Enhance Anti-Inflammatory Macrophage Responses
Source: Biol Trace Elem Res. 2026 Jul 3;204(9):7358–72. doi: 10.1007/s12011-026-05192-5 (PMC13437566; doi:10.1007/s12011-026-05192-5)
Supplement: Supplementary file 5 [file 12011_2026_5192_MOESM5_ESM.pdf]

Figure S5.

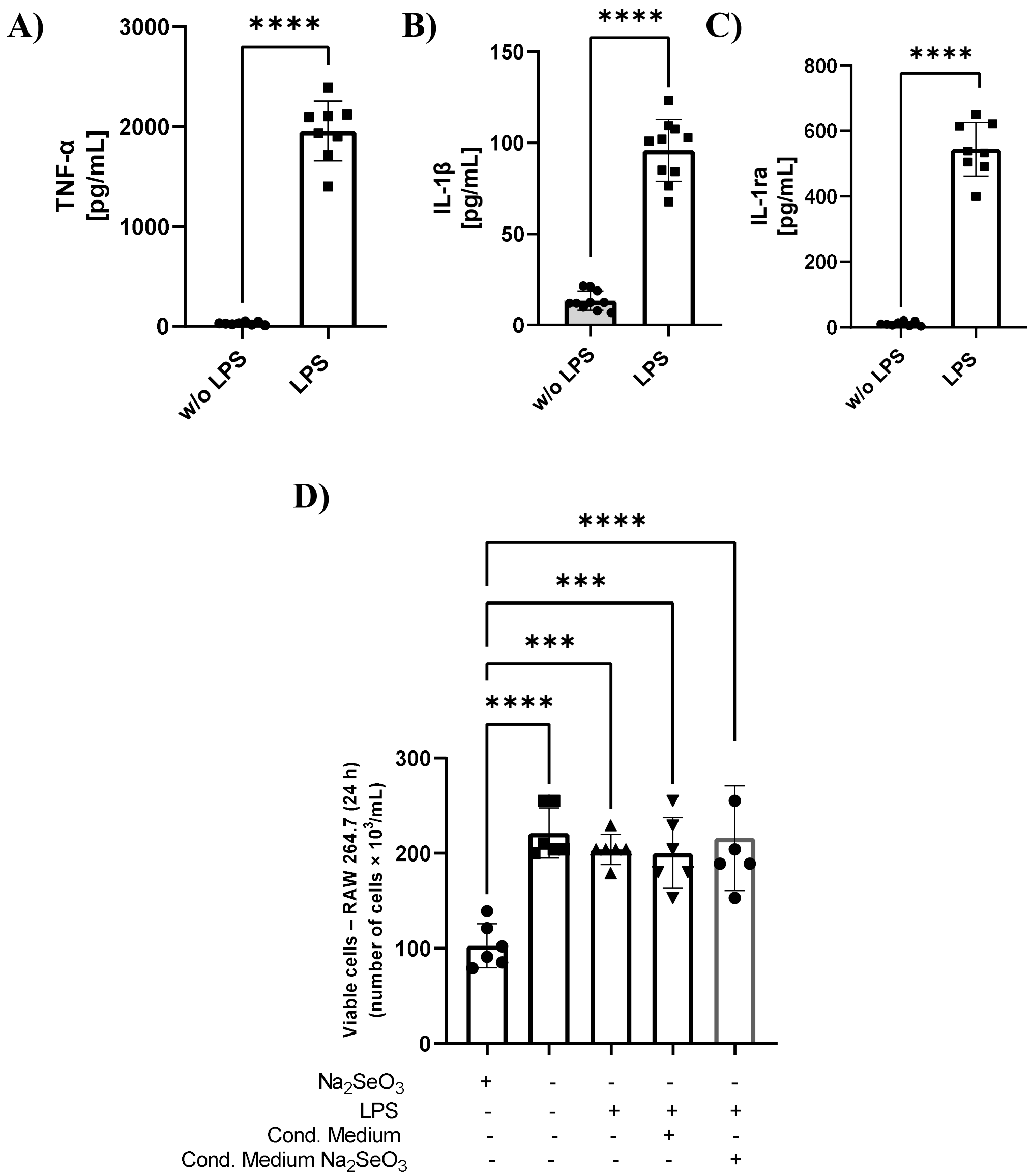

Figure S5. LPS-induced inflammatory response and viability in mono-macrophage cells**.** Mono-macrophage cells were incubated for 24 hours with or without LPS stimulation. The production of (**A**) TNF-α, (**B**) IL-1β, and (**C**) IL-1ra by macrophages was measured. (**D**) The viability of mono-macrophage cells stimulated or not with LPS and incubated or not with conditioned media was assessed. Data are expressed as mean ± SD. $n = 6$. (****$p < 0.0001$).
